# A Hierarchical Neuronal Model for Generation and Online Recognition of Birdsongs

**DOI:** 10.1371/journal.pcbi.1002303

**Published:** 2011-12-15

**Authors:** Izzet B. Yildiz, Stefan J. Kiebel

**Affiliations:** Max Planck Institute for Human Cognitive and Brain Sciences, Leipzig, Germany; Indiana University, United States of America

## Abstract

The neuronal system underlying learning, generation and recognition of song in birds is one of the best-studied systems in the neurosciences. Here, we use these experimental findings to derive a neurobiologically plausible, dynamic, hierarchical model of birdsong generation and transform it into a functional model of birdsong recognition. The generation model consists of neuronal rate models and includes critical anatomical components like the premotor song-control nucleus HVC (proper name), the premotor nucleus RA (robust nucleus of the arcopallium), and a model of the syringeal and respiratory organs. We use Bayesian inference of this dynamical system to derive a possible mechanism for how birds can efficiently and robustly recognize the songs of their conspecifics in an online fashion. Our results indicate that the specific way birdsong is generated enables a listening bird to robustly and rapidly perceive embedded information at multiple time scales of a song. The resulting mechanism can be useful for investigating the functional roles of auditory recognition areas and providing predictions for future birdsong experiments.

## Author Summary

How do birds communicate via their songs? Investigating this question may not only lead to a better understanding of communication via birdsong, but many believe that the answer will also give us hints about how humans decode speech from complex sound wave modulations. In birds, the output and neuronal responses of the song generation system can be measured precisely and this has resulted in a considerable body of experimental findings. We used these findings to assemble a complete model of birdsong generation and use it as the basis for constructing a potentially neurobiologically plausible, artificial recognition system based on state-of-the-art Bayesian inference techniques. Our artificial system resembles the real birdsong system when performing recognition tasks and may be used as a functional model to explain and predict experimental findings in song recognition.

## Introduction

Songbirds are able to repeat the same, often complex songs with amazing precision. When male birds sing to a female repeatedly, there is on average a 1% temporal deviation across the whole song [1], [2]. This combination of complexity and precision is remarkable. Studying the neuronal basis of birdsong generation may lead to an understanding of the mechanism underlying how sequences of song syllables are expressed as complex and temporally precise sound wave modulations. More generally, such a mechanism may also be useful for understanding how action sequences at a relatively slow time-scale (e.g. the words in a sentence) can be generated by a neuronal system while a high degree of precision is maintained in the output at a fast time-scale (e.g. the sound wave modulations necessary to form speech sounds).

Recent findings [1]–[3] have shown that the song generation mechanism in birds is hierarchical where neurons in one particular high-level structure, HVC, fire in a specific sequence with high temporal precision and drive neurons in the lower level structure RA (robust nucleus of the arcopallium).

Female birds, at which the songs are typically directed, are expert in registering variables like the speed of the song and the precision and the repertoire of the singer [4]–[8]. Unfortunately, the study of song recognition is more challenging than song generation because experimental indicators for recognition, such as the subsequent behavior of a female bird, are more difficult to measure than indicators for song generation. This has led to a long list of experimental and theoretical findings on song generation and learning while the mechanisms of song recognition remain relatively elusive.

Here, we propose that the functional mechanism of song *recognition* can be obtained from the song *generation* mechanism. The basic idea underlying this novel modeling approach is that female birds are optimal in song recognition because their mating choice critically depends on the optimal recognition of valuable features of the male which are revealed by subtle indicators in his song. Similarly, male birds should be able to distinguish the songs of their neighbors from the songs of strangers to protect their territories [9], [10]. Using a recently established Bayesian inference technique for nonlinear dynamical systems [11], we can emulate this optimal recognition: the key ingredient is a generative model (a nonlinear dynamical system) which can generate a specific song. Usually, generative models for complex sensory dynamics, such as the sound wave or spectrum of birdsong, are difficult to derive because it is hard to describe a complex multi-scale structure like birdsong using only differential equations. Fortunately, since the hierarchical birdsong generating system is so well-studied, parts of such a model already exist, in particular at the level of the HVC, RA and vocal tract dynamics [12]–[18]. We have combined these parts into a coherent whole, guided by key experimental results, to form a generative model that can play complex songs. In particular, we combined sequence-generating dynamics, attractor dynamics and a model of vocal tract dynamics [17] in a three-level, hierarchical nonlinear dynamical system. This dynamic model is based on neuronal rate models, thereby describing the biological system at a mesoscopic level. We then used Bayesian inference to derive another set of hierarchical, nonlinear differential equations (recognition system) which is, by way of construction, Bayes-optimal in recognizing this song and can be compared to the real birdsong recognition system. To do this, we exposed the agent to several tasks and found that the agent's dynamics and performance were reminiscent of song recognition in real bird brains in aspects such as sensitivity to speed changes [19] and song perturbations [20], [21]. Thus, by harnessing rich experimental and theoretical results in birdsong generation, we were able to derive a novel, functional model of birdsong recognition. We discuss the experimental evidence that the identified mechanism is indeed used for song recognition by birds. We suggest that the present model may be useful for understanding the functional and computational roles of auditory recognition areas. In addition, the identified recognition mechanism can be used as a novel machine learning tool to recognize sequential behavior from fast sensory input, e.g. in artificial speech recognition.

## Model

In this section, we will briefly summarize relevant experimental findings, motivate and describe the present model for birdsong generation and briefly give the mathematical details.

**Figure 1:**
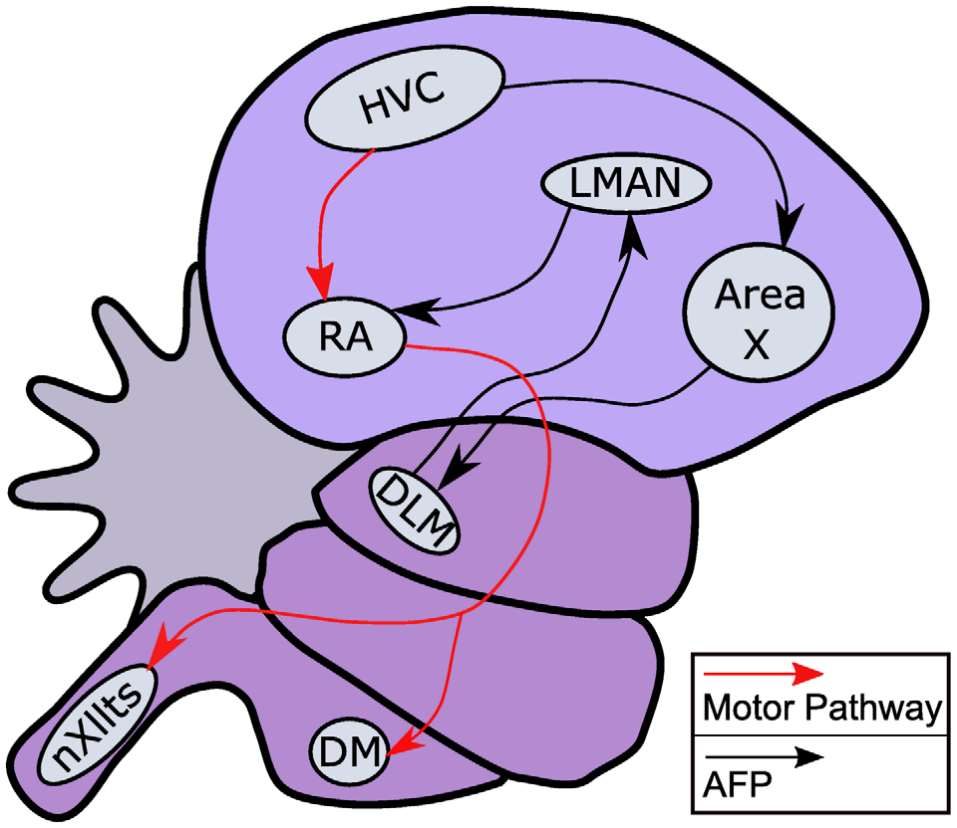
The schematic diagram of a songbird brain with the motor pathway (red arrows), which is considered in the model of song generation, and the anterior forebrain pathway (AFP, black arrows). In the motor pathway RA neurons that are driven by the HVC control the motor (nXIIts innervates the syrinx) and respiratory (DM) areas. The anterior forebrain pathway communicates with the motor pathway through the LMAN area that provides direct input to the RA region. Abbreviations: DLM, nucleus dorsolateralis anterior, pars medialis; DM, dorsomedial nucleus; HVC, a letter based name; LMAN, lateral magnocellular nucleus of the anterior nidopallium; nXIIts, tracheosyringeal portion of the nucleus hypoglossus; RA, robust nucleus of the arcopallium. Adapted from [87].

A birdsong consists of small units called notes (analogous to phonetic units in speech) which can be grouped together to form syllables [22]. A combination of identical or different syllables forms motifs. This hierarchical structure of song units is produced by two highly specialized song pathways (Figure 1, see [23] for a review). In the motor pathway, the forebrain nucleus HVC includes specific neurons called HVC_(RA)_ that project to nucleus RA. RA neurons innervate the vocal and respiratory nuclei to produce vocal output. The anterior forebrain pathway is involved in learning new songs and producing variability for the song structure [24].

**Figure 2:**
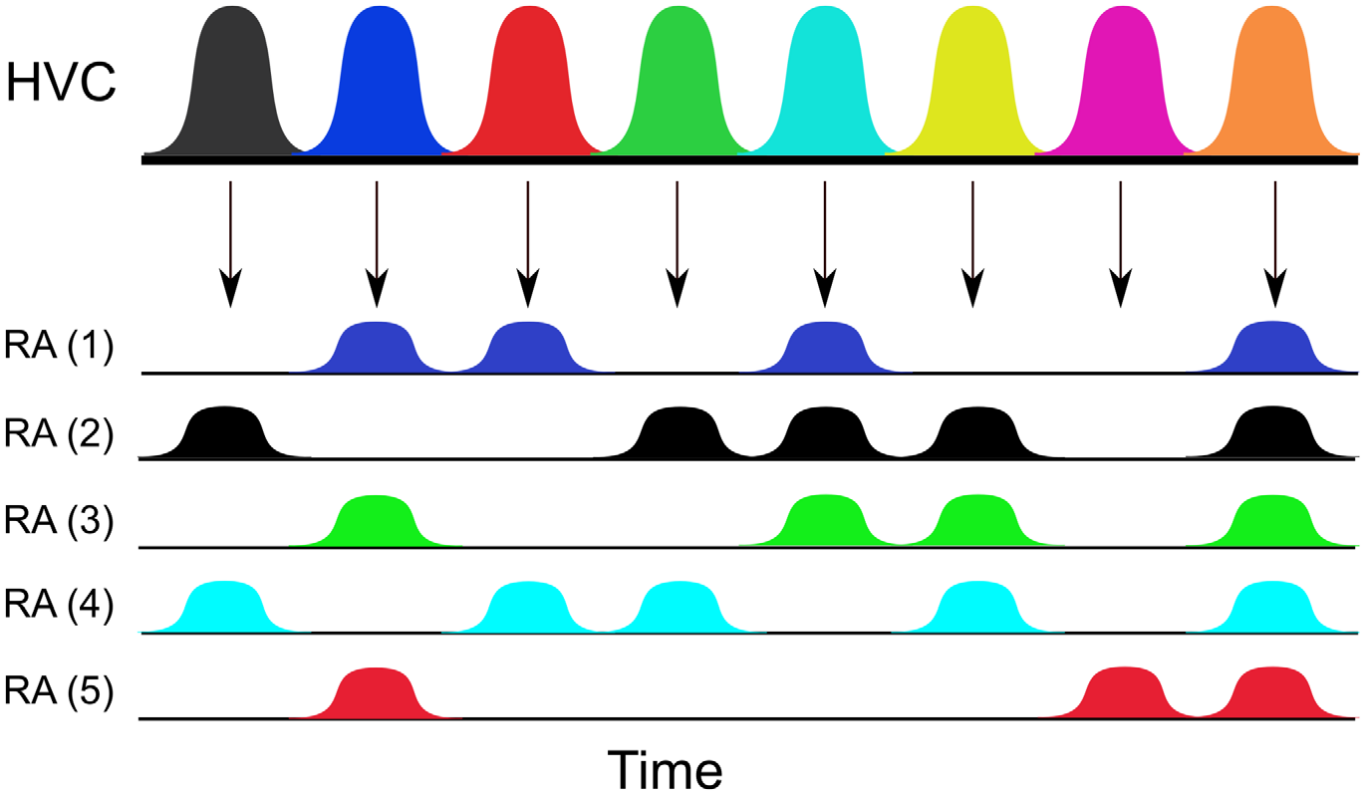
The scheme of HVC and RA dynamics. Five RA ensembles are controlled by eight sequentially activated HVC_(RA)_ ensembles. The horizontal axis denotes time and the arrows describe the specific HVC ensemble that activates the corresponding RA ensembles. The color scheme matches the dynamics shown in Figure 5. The part of the song obtained from the first three RA-patterns (i.e., ensemble combinations 2–4, 1-3-5 and 1–4) is shown as a sonogram in Figure 3.

Our modeling approach is based on the following key experimental observations: During birdsong generation, HVC_(RA)_ neurons fire sequentially at temporally precise moments where each element of this sequence fires only once during the song to control a group of RA neurons [2], [3], [25]. This suggests that bursting HVC_(RA)_ neurons select and drive the activity of subsets of RA neurons [25]. In particular, each RA neuron can be driven by more than one HVC_(RA)_ neuron [26], see Figure 2.

How can one model such a mechanism? There have been several approaches to model the sequential activation of HVC_(RA)_ neurons using single neuron models [12]–[15]. Here, we follow an alternative way by capturing the neuronal mass activity using firing rate models, i.e. we consider model neurons that can be thought of as the synchronized firing activity of an ensemble of neurons. This is motivated by experimental evidence suggesting that there are about 200 co-active HVC_(RA)_ neurons at a specific time during song generation [25]. One of the well established ways for modeling the sequential activation of neuronal ensembles is the winnerless competition using Lotka-Volterra type dynamics [27], [28]. This approach aims at modeling activity at a mesoscopic level, e.g. activity that may be expressed in local field potentials.

Another benefit of using ensemble dynamics appears at the RA level where each ensemble controls the vocal tract muscles in a specific way. Different than HVC_(RA)_ ensembles, one or more RA ensembles can activate simultaneously (synchronize) [25] (Figure 2). We hypothesize that the complex sound wave modulations that can be observed in many birdsongs are generated by this network of RA ensembles using spatiotemporal coding (see also [26]). This coding requires the activation of different sets of RA ensembles (spatial coding) when the proper signals are received from the corresponding HVC_(RA)_ ensembles (temporal coding). This spatiotemporal coding can be modeled with network states which are driven from one attractor to another where each of these attractors specifies the currently active RA ensembles. In other words, when HVC_(RA)_ ensembles undergo sequential activations, the RA level is driven from one attractor to the next. Such networks with attractor dynamics (so called Hopfield networks [29]) can encode a large number of potential attractors because the forcing input from the HVC level effectively recombines subsets of RA ensembles in distinct assemblies.

Note that since the activity of each unit represents the average firing rate of an ensemble of neurons, some features of neural activity at the level of individual neurons are not considered. Here, we focus on capturing the two key features of the hierarchy, which are the sequential firing of HVC ensembles and the spatiotemporal coding at the RA level. Therefore, the choice of parameters in the computational model below are motivated by capturing the specific dynamics inferred by experiments [25].

**Figure 3:**
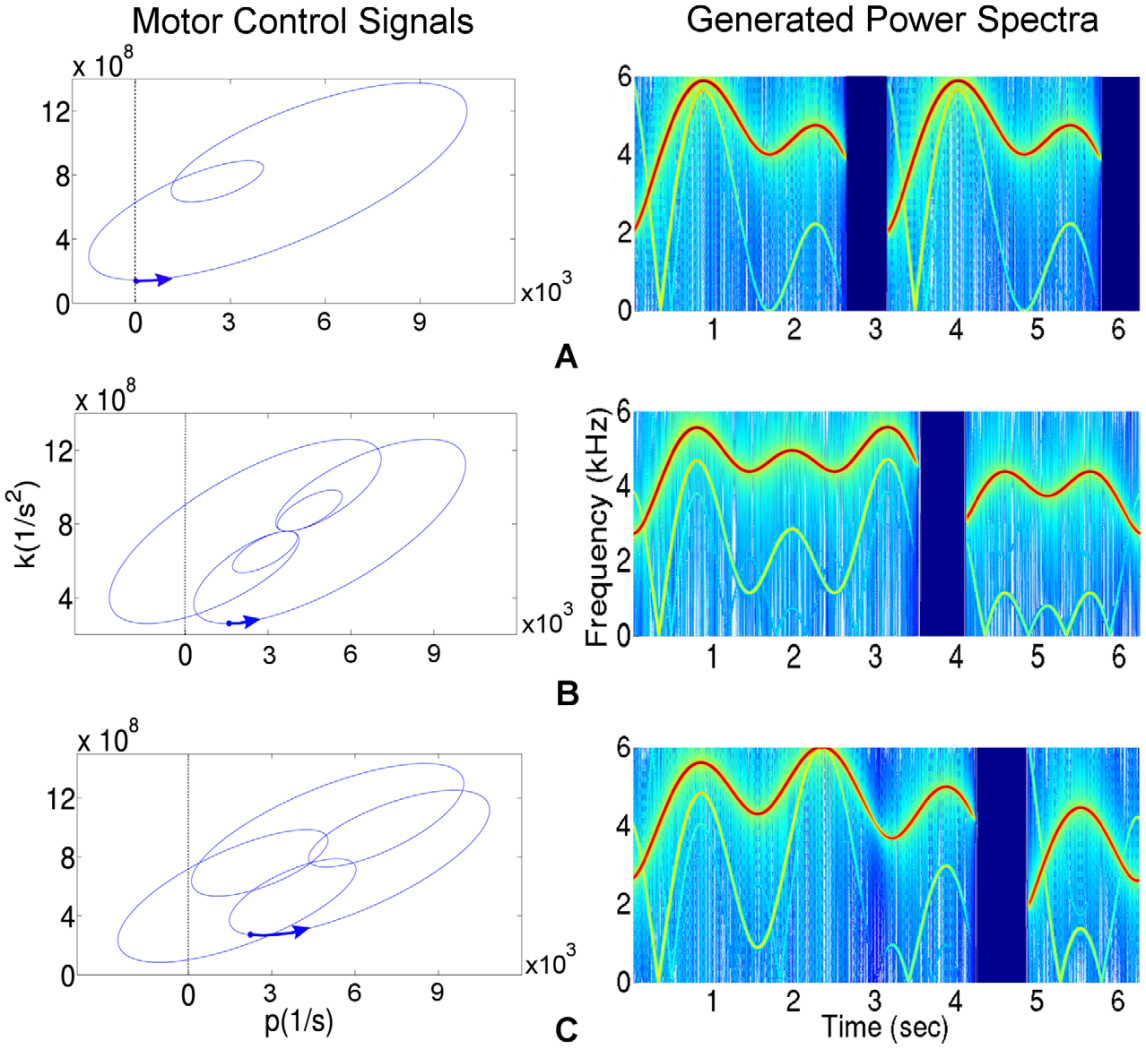
Motor control signals and resulting power spectra generated by the model. Left: The motor control signals are obtained by a linear combination of sine waves (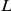) with 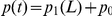 (*x*-axis) and 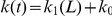 (*y*-axis) where 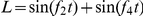 in (**A**), 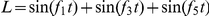 in (**B**) and 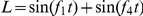 in (**C**). Right: These 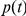 and 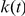 dynamics are used in the syrinx equations (4) to obtain sound waves with the corresponding sonograms (time (sec) vs. frequency (kHz)). 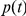 and 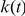 control the amplitude and frequency of the sound waves, respectively. When 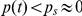, no phonation is produced (mini-breaths, [44]). The fluctuations in the fundamental frequencies in the sonograms on the right can be traced by moving in counter clock-wise direction on the ellipse-like curves on the left starting from the blue arrows at 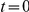.

At the lowest level, we map the dynamical RA states onto motor neurons. To do this, we compute linear combinations of oscillators at different frequencies which represent the effect of currently active RA ensembles and create dynamical control signals (Figure 3) for a model of the vocal organ, the syrinx [17]. This mathematical model of the syrinx has been used previously to model several birdsongs [30], [31].

**Figure 4:**
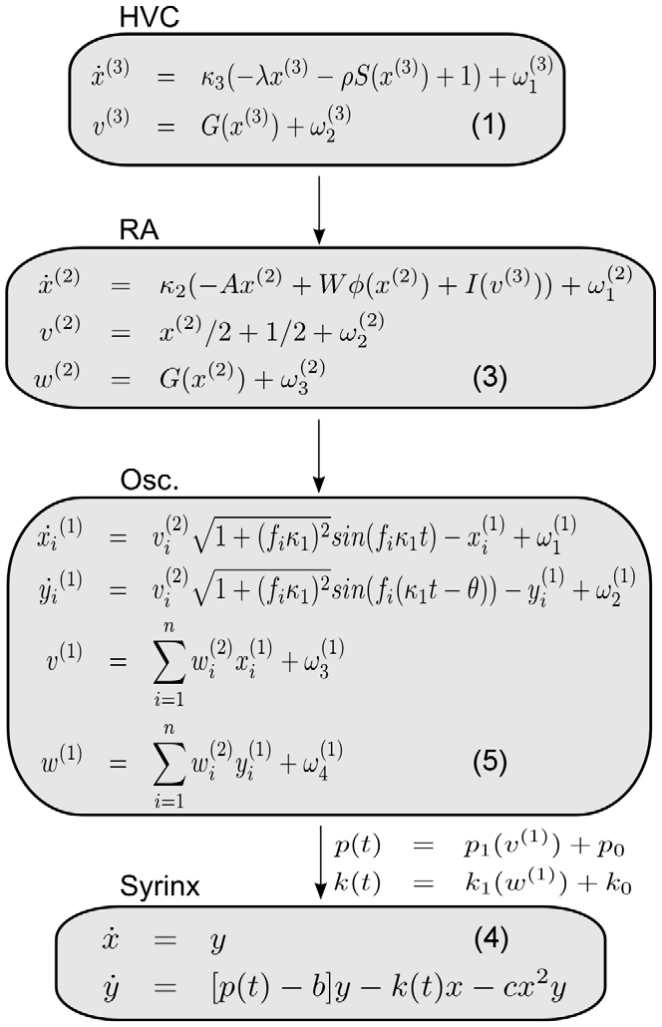
Summary of nonlinear differential equations (1), (3), (4) and (5) (see Text S1 for Eq. (5)) that are used in the hierarchical model for birdsong generation. Notice that the output at each level is used as an input to the lower level. Typical dynamics of HVC, RA and oscillator (Osc.) levels are given in Figure 5A, 5B and 5C, respectively. Finally, the output of the oscillator level is used in the syrinx equations to produce appropriate sound waves (Figure 6). See Table 1 for the parameters.

In summary, the present three-level hierarchical model generates sequences at its top (HVC) level, which are transformed into sequences of multi-dimensional attractors at the RA level. Each of these attractors encodes a mixture of oscillations. These oscillatory dynamics enter a syrinx model as a control signal to produce a birdsong sonogram. In the following, we describe the equations used at each level in detail (see Figure 4 for an overview). The Bayesian recognition of dynamics generated by this birdsong model is described at the end of the section.

### HVC Sequential Dynamics

Lotka-Volterra equations are well known in population biology to describe the competition between species [32]. Rabinovich et al. (see [33] for a review) applied this idea more generally to neuronal dynamics under the name of winnerless competition, see [28] and [34] for applications. In the following, we will describe how one can apply this idea to model sequential HVC activity by a nonlinear dynamical system. In the winnerless competition setting, there are 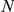 equilibrium points which are saddles of a nonlinear dynamical system. Each of these equilibrium points has a single unstable direction and all other directions are stable. One can think of these saddle points as the beads on a string where the unstable manifold of one saddle point is the stable manifold of the next saddle point and this sequence continues in a circular fashion forming a heteroclinic chain. Under some conditions [27], this sequence is stable, i.e. a solution of the system that starts from a neighborhood of the chain, stays in this neighborhood at all times while traveling through all saddle points. This stable sequential behavior is what we exploit to model the experimentally established sequential activities of HVC_(RA)_ ensembles at the highest level. As the solution of the system moves along the string, it visits all saddle points, i.e. each HVC_(RA)_ ensemble, one by one thereby activating each ensemble for a brief period until it is deactivated as the next ensemble becomes active.

These dynamics can be obtained from a neural mass model of mean membrane potential and action firing potential [35], reviewed in [33]. We use the equations:
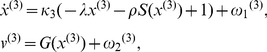
(1)where 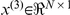 is the hidden-state vector (e.g., mean membrane potentials) at the third (HVC) level, 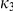 and 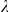 are scalars, 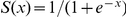 is the sigmoid function applied component-wise and 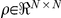 is the connectivity matrix with entries 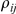 giving the strength of inhibition from state 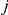 to 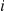. The second equation describes the output vector (or causal-state vector; e.g., neural firing rates) 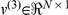 where 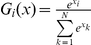, 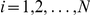, is a normalizing function. We also add normally distributed noise vectors 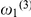 and 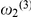 to render the model stochastic. With an appropriately chosen connectivity matrix, one can obtain a system with 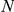 saddle points forming a stable heteroclinic chain [27]. For the entries of the connectivity matrix, one chooses high inhibition from the previously active neuron to the currently active neuron and low inhibition from the current active neuron to the next neuron which will become active:
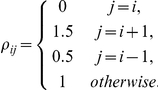
(Here 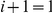 when 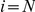 and 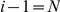 when 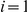).

**Figure 5:**
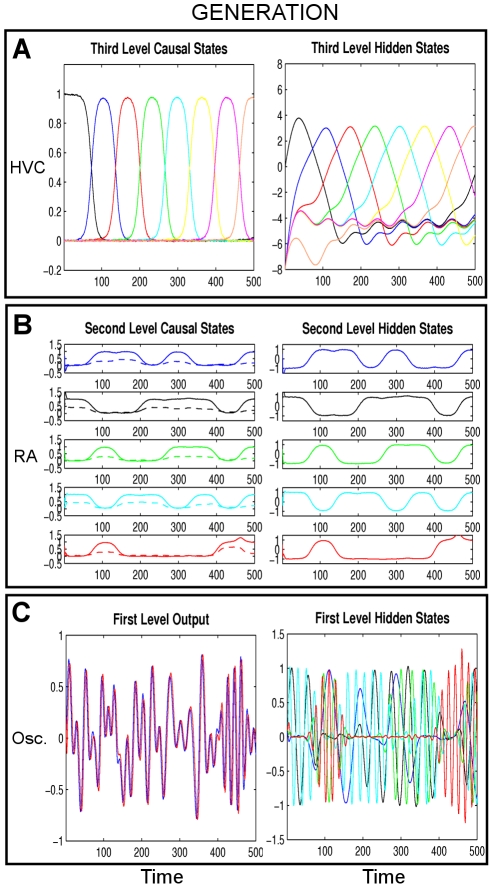
Generated dynamics for the first simulation ‘Ideal communication’. The causal states are shown on the left and hidden states on the right with arbitrary units both in time and neuronal activation. There are three levels: **A**) HVC (third) level, Eq. (1), **B**) RA (second) level, Eq. (3) and **C**) Oscillator (first) level, Eq. (5) in Text S1. At the third level, there are eight HVC ensembles (each represented with a different color) which are activated for a short amount of time to control the dynamics of the five RA ensembles, see also Figure 2. At the second level (left column), the solid lines represent 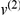 and dashed lines represent 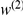, see Eq. (3). At the first level (right), we only show 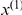 since 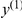 is a shifted version of 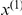, see Eq. (5) in Text S1. At the first level (left), the blue line is 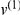 and the red line is 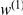 (which are mostly overlapping because of phase-locking). These output dynamics control the syrinx to obtain synthetic birdsong (Figure 6).

Note that, theoretically, one can generate arbitrarily long sequences of HVC activation using the above connectivity matrix. The stability region around the heteroclinic chain will persist for much longer sequences than the one modeled here. For our illustrative simulations described below, we use 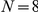, i.e., there are 8 HVC_(RA)_ neuronal ensembles but the model works robustly with more HVC_(RA)_ ensembles as well (see Figure S1). A real bird brain has many more HVC_(RA)_ ensembles but here we are interested in presenting a general mechanism for which a small selection of HVC and RA ensembles is sufficient. See the third level dynamics in Figure 5A for typical dynamics generated by this system.

We control the dynamics of RA ensembles by letting the *k*th HVC_(RA)_ ensemble send a signal 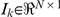 to the lower level during its activation time. See the next subsection for details of how this signal vector is computed. The total signal sent to the lower level by all HVC_(RA)_ ensembles at any time is a linear combination of the 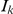
's: 
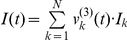
 where 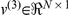 is the output vector in Eq. (1). Note that for typical sequential dynamics at the HVC level, except for the transition times, only one entry in 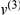 is active (i.e., only one entry is close to 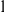), see Figure 5A.

### RA Attractor Dynamics

Experimental findings suggest that activation of different HVC_(RA)_ ensembles drives the activation of different *combinations* of RA ensembles [25]. In the present model, we capture this by forming a network of RA neuronal ensembles whose dynamics converge to one of several attractors depending on the input from the HVC level (see Figure 2). This means that the RA level receives input from the HVC level and produces output which encodes the level of activity of each RA ensemble at a given time. Since we are working with continuous systems, the notion of attractors comes up naturally as the RA ensemble activity flows from one activity pattern to another one. To achieve this smooth flow between RA attractors, we have to use a nonlinear network because otherwise the RA level would simply copy the dynamics of the HVC level. Note that, similar to the HVC level, the intrinsic neuronal dynamics of the RA are not established well experimentally. In this situation, we aim at describing underlying population dynamics which give rise to the experimentally observed key features of RA dynamics [25]. To implement these dynamics, we use a well-established type of an attractor-based network described by Hopfield [29]. Hopfield networks have been mostly used as a model of associative memory where each memory item is encoded by an attractor. When such a system receives noisy sensory input, i.e. it is started at some nearby initial state, it evolves to an attractor (the memory to be retrieved) [29], [36]. Here, we use this idea to encode the activities of RA ensembles by attractors. As the attractor of the network changes continuously due to driving HVC input, the activities of RA ensembles also changes such that some RA ensembles activate and some others deactivate. This gives us the spatiotemporal coding that drives the syrinx dynamics described in the next subsection. We use a Hopfield network with *asymmetric* connectivity matrices [37]–[39] given by the following equation:
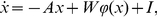
(2)where 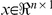 is the ensemble state vector with 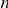 ensembles, 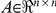 is a diagonal positive matrix which governs the rate of change of each ensemble's state, 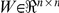 is a synaptic connectivity matrix with entries 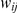 denoting the strength of connection from ensemble 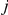 to ensemble 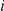, 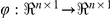 is the activation function which we take as *tanh* function applied component-wise and 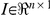 is the direct input from the HVC level. This equation is similar to Eq. (1), i.e. both are continuous-time recurrent neural networks, but in Eq. (2) we have an additional input vector and different conditions on the connectivity matrix as described below. In addition, the use of the nonlinear activation function 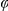 brings more plausibility to the network, as compared to linear dynamics, since the effect of one RA ensemble to another one does not increase linearly but saturates.

The input vector 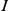 should be chosen such that RA ensembles get quickly attracted to a desired attractor. An attractor means that a subset of the RA ensembles are ‘active’ (taking the value 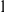) while all other RA ensembles are inactive (taking the value 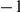). The goal is to establish conditions for the network in Eq. (2) to have a globally asymptotically stable equilibrium point (a vector 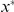 that makes the right hand side of Eq. (2) zero and attracts all the solutions regardless of the initial state). These conditions and the proper choice of 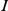 for the desired attractor have been described in [38] and [40] (see Theorem 1 in Text S1).

Using this technique, we can employ a small number of RA ensembles to encode a larger number of desired attractors to control the lowest level, the motor output. Each HVC_(RA)_ ensemble provides a different 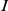-vector to the RA level thereby driving the RA ensembles into a unique attractor. The application of this is that each RA level attractor will drive the motor output in a specific way thereby producing a different part of the song. We obtain the equations for the second level by combining the Hopfield network, Eq. (2), with the two output equations (state vectors) 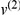 and 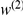 where superscripts denote the specific level of a variable:
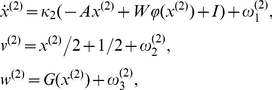
(3)where the exact form of the connectivity matrices 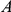, 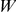 and the HVC input vector 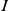 are described in Text S1, 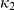 is a scalar and 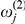 are normally distributed noise vectors. 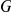 is the normalizing function as in Eq. (1). Note that 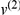 squeezes the entries of 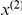 into the interval 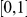 but 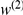 may return values smaller than 1 since more than one entry of 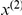 can be active (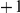) at a given time. The vectors 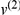 and 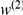 carry the output of the second level to the first level (oscillator level) as described in the next subsection.

In the present model, we use 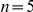 (i.e., five RA ensembles, Figure 5B). Note that there are 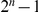 different ways to activate 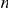 RA ensembles to produce motor output. We use 7 of these 31 combinations (one occurring twice) in Figure 5 for generation of an example song (with 8 HVC_(RA)_ ensembles at the higher level). In the figures, we used arbitrary units for both time (x-axis) and neuronal activation (y-axis) because we consider neuronal ensembles.

### Model of the Avian Vocal Organ

The avian vocal organ, the *syrinx*, is located at the base of the trachea (windpipe) where the trachea divides into the bronchi. A set of soft tissues within the syrinx, the *labia*, which are similar to human vocal folds, oscillate with the airstream propelled from the air sacs. Sound waves generated from these oscillations propagate through the trachea and beak. Therefore, these sound waves are modeled as the oscillations of the labia which are produced by the vocal control signals: the air sac pressure, 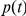, and the stiffness of the labia, 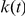. Such a mathematical model of the vocal fold oscillations was first given by Titze [41], and similar oscillations were experimentally observed in the bird syrinx [42]. A simplified version of this model (using a polynomial approximation for the nonlinear dissipation) can be given as follows [17]:
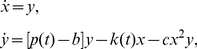
(4)where 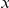 is the position of the labia from the midpoint of the syrinx, 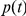 denotes the air sac pressure, 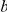 is the linear dissipation constant, 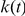 is the stiffness of the labia and 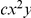 is a dissipation term to prevent the big amplitude oscillations when the labia meet each other or the walls of the syrinx [43]. The fundamental frequency of the sound wave increases or decreases proportional to 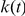. Note that there is a critical value 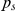 for the pressure such that if 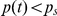, no phonation is produced. This region in the parameter space corresponds to the mini breaths between syllables [44]. Using this simple model, one can obtain accurate copies of some birdsongs such as canary [30], chingolo sparrow [17], white-crowned sparrow [31] and cardinal [45] by choosing appropriate vocal control signals for the syrinx (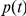 and 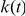) as described next.

### Vocal Control Signals

Oscillators as in the present model have been widely used to model movement patterns in animals and humans. Central pattern generators are a well-known example of neural networks that are used to generate periodic motor commands such as locomotion [46]. We use the same principle here, and use five oscillators with different frequencies (one for each RA ensemble) to let the RA dynamics drive the vocal output (syrinx) mechanism, see Eq. (4). Note that it is experimentally not well established how the RA level controls the syrinx muscles; our approach is a natural extension of the phenomenological syrinx model described above [17]. The main point here is that the oscillator level (first level) is assumed to generate mixtures of oscillations (hidden states) where the RA level activity at the supraordinate level controls which oscillations should be produced at a given time. Each RA ensemble is assumed to control the activity of a single oscillator at the level below Therefore, the spatiotemporal coding of the RA level is transformed into the oscillatory activity of the first level which generates the final *p(t)* and *k(t)* dynamics necessary to control the syrinx.

As oscillators, we choose simple sine wave equations where the lowest frequency oscillator 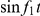 corresponds to the slowest-changing dynamics of the birdsong. We choose the remaining four oscillators such that their frequencies are integer multiples of this first oscillator's frequency (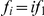): 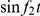, 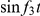, 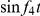 and 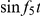. Each one of these sine waves represents faster changing dynamics of the song; 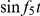 being the fastest. In this way, we can model effects in the birdsong which express themselves on different time-scales.

We include these five oscillators in the present model at the first level, where each of the five ensembles at the RA level controls the amplitude of one of the oscillators (through 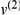, Eq. (5) in Text S1). The observable output is obtained by taking a linear combination of these amplitude-modulated sine waves. To drive the vocal model appropriately, we produce two outputs 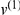 and 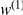 (the second output is simply a time-shifted copy of the first one), which are involved in producing air sac pressure *p(t)* and the stiffness of the labia *k(t)*. 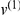 and 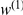 are described in detail in Text S1.

**Figure 6:**
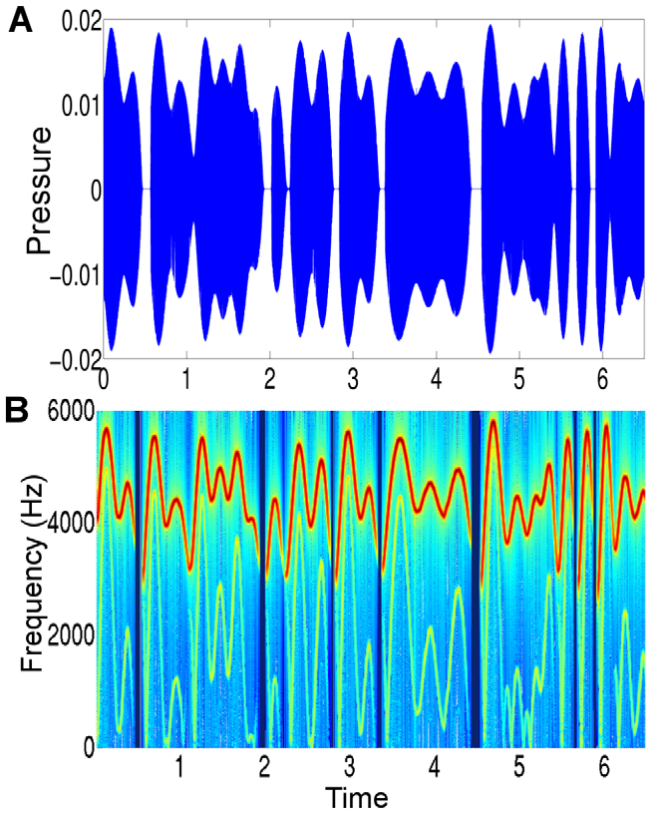
The sound wave and sonogram of a generated song. We plugged the air sac pressure (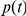) and stiffness (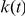) parameters obtained from the first level output of the generative model (Figure 5C) into the syrinx equations (4). **A**) The solution of the syrinx equations, i.e. 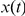 in Eq. (4), arbitrary units. The mini-breaths where no phonation is produced can be clearly observed. **B**) The sonogram of the soundwave in A (time (sec) vs. frequency (Hz)) is given with a sampling frequency of 12000 Hz. The first ∼3 sec of this sonogram can also be viewed in separate chunks in Figure 3.

Laje et al. [31] chose 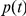 and 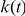 to form several ellipses in the 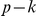 parameter space where each ellipse corresponds to a different syllable. However, this parameterization may not support complicated syllables which have more fluctuations on the sonogram. Here, we extend their model to increase the complexity of the generated songs by using the linear combination of different frequency sine waves (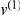 and 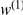 described above) to parameterize these two functions and obtain a variety of ellipse-like curves in the 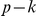 parameter space (see Figure 3):
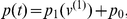


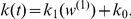
where 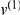 and 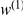 are the outputs of the first level and the scalars are given in Table 1. These ellipse-like curves can be plugged into Eq. (4) to obtain synthetic birdsongs. See Figure 6 for the sonogram obtained using the first level output of the generation process shown in Figure 5C. The sonogram can be played and is reminiscent of a birdsong (Audio S1). Note that in the real system, longer HVC_(RA)_ sequences would be required to produce a song with 6.5 seconds duration since HVC_(RA)_ bursts last only about 6–10 ms [25]. Here, we assume that each HVC_(RA)_ ensemble in the model is a collection of at least 80 HVC_(RA)_ neurons that fires sequentially and controls the timing of the song for about 800 ms.

**Table 1:** Variables used in the generative and recognition models.

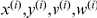	Hidden states, 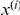 , 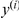 and causal states, 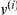 , 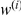	
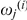	Normally distributed noise vectors at the 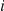 th level	
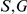	Sigmoid ( 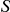 ) and normalizing ( 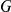 ) functions	
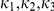	Rate constants: 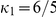 , 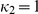 and 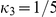	
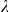	Decay rate: 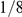	
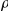	Connectivity matrix of the HVC level	
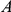	Diagonal matrix with diagonal 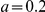	
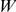	Connectivity matrix of the RA level	
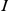	Direct input from the HVC to the RA level	
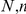	Number of HVC ( 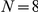 ) and RA ( 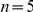 ) ensembles	
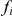	Angular frequencies: 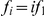 where 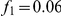	
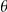	Phase-shift: 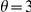 time units.	
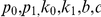	Syrinx control parameters:	
	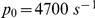	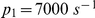
	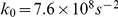	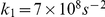
	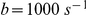	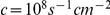
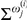	Covariance matrices for the noise in generation: 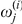	
	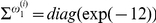	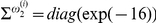

This table lists the variables of the equations shown in Figure 4 and Eqs. 1 to 5.

### Online Bayesian Recognition

In this subsection, we will briefly describe the present recognition scheme for the generated songs. This scheme is a model of vocal communication between conspecific birds but may also serve as a functional model to explain experimental findings along the auditory processing pathway which is less understood than the song pathway. Here, we describe a potential mapping of this Bayesian inference framework to neuronal dynamics at a population level, see [47], [48]. The inference is based on hierarchical message passing and implements a predictive coding scheme for dynamics. As summarized below, all the update equations of the recognition system (to reconstruct the hidden states) consist of differential equations (as in the generation model) and therefore may be implemented by neuronal populations and their network interactions via forward, backward and lateral connections [47], [48].

How can a bird recognize a conspecific's song and decode the information contained in the song? This decoding is important as it is known that female birds select their mates according to criteria such as the complexity of the male's repertoire [7] or the precision of the vocal performance [8] and they show preference for the songs of their mates or fathers compared to the songs of strangers [4], [49], [50]. In general, this suggests that listening birds may have certain expectations (priors) about the type of the song they expect to hear. In general, we assume that listening birds have internal models for the songs they have learned before and the generative model of the heard songs should fit to this internal model.

Using this concept, we model optimal recognition using Bayesian inference for hierarchical, nonlinear dynamical systems [47].

**Figure 7:**
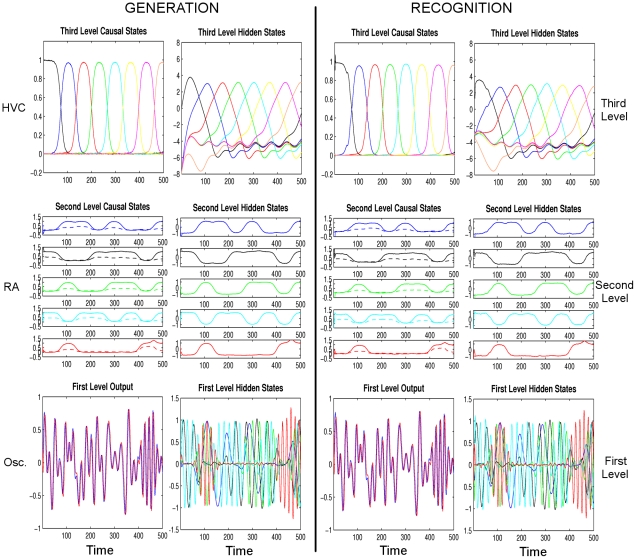
First simulation ‘Ideal communication’: The dynamics of song generation (left two columns) and song recognition (right two columns) with arbitrary units. The format and the generated dynamics are the same as shown in Figure 5. The recognition scheme receives only the output of the first level (bottom left) and reconstructs states at all levels using the online Bayesian inference scheme. It can be seen that the reconstruction is successful as there are only tiny deviations between the true (left) and the reconstructed (right) dynamics.

For the sensory input, we assume that the vocal control signal 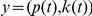, given the sound wave, can be readily extracted by the listening bird (agent) from the spectrotemporal dynamics, see Figure 3. Here, we consider the *p(t)* and *k(t)* dynamics, in the recognition step, as an abstract representation of the song spectrum and therefore a phenomenological approximation to the highly nonlinear features of the singing bird's syrinx. This means that we assume that the listening bird has access to these dynamics via some low-level recognition process. For the present implementation of the inference framework, the full inference from the soundwave (Figure 7) would currently be computationally too expensive because this would require a high temporal resolution, e.g. at 12 kHz, and long time-series. However, once an optimized (parallel) implementation of the present framework becomes available, the present model can be extended in a straightforward fashion to model recognition that receives a soundwave as sensory input by adding another level that transformed the *p(t)* and *k(t)* dynamics to soundwaves.

Given this vocal control signal, we infer the spatiotemporal RA dynamics and the sequential HVC_(RA)_ dynamics. The proposed Bayesian inference scheme provides, under some assumptions, optimal inference to decode the RA and HVC_(RA)_ dynamics, i.e. to recognize the hidden messages embedded into the vocal control signal.

The mathematical description is provided below and can be conceptualized as follows: At each time step *t*, the recognition system receives sensory input, here the current amplitudes of the *p(t)* and *k(t)* dynamics. Like the generative model, the recognition system has three levels as well. Each of these three levels consists of interacting neuronal populations, which encode predictions, i.e. expectations, about how their internal dynamics will evolve during a song. At the same time, each level receives input from the subordinate level. For the first level, this is the sensory input, which is compared with the internal prediction. The prediction error is forwarded to the second level, where again predictions are used to generate prediction errors, which are forwarded to the third level. Critically, each level adjusts its internal predictions to minimize its prediction error weighted by the prior precision of the internal prediction. At each level, the updated predictions are sent to the subordinate levels to guide their internal predictions by higher level predictions. In summary, each level minimizes its prediction error by a fusion of internal dynamics with top-down (predictions) and bottom-up (prediction error) messages. The overall result is that a listening bird fuses its dynamic and hierarchically arranged expectations about a song with the actual sensory input. Importantly, due to this dynamic fusion, the recognition is robust against deviations from its expectations by explaining away errors of the singing bird by internal precision-weighted prediction error. The derivation of the update equations to achieve Bayes-optimal online recognition solutions is non-trivial, see Friston et al. [11]. Note that this modeling approach implies that generation and recognition models are fundamentally different from each other in the sense that generation is a top-down process where recognition consists of both top-down and bottom-up processes. Although some of the computations in the generation and recognition model are the same and may provide a computational explanation for mirror neuron accounts [51], this is not a central issue in the present paper and we assume here that recognition is performed by neuronal populations different from those that generated the song. Clearly, this remains an open question that can only be settled experimentally.

For sensory input 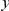 and a given model 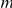, the probability 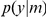 is called the *model evidence* or *marginal likelihood of* 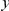 and is an important quantity for model comparison among different models. In our case, 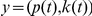 is the vocal control signal for the syrinx which we take as the input and the model 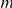 (Figure 4) includes all the parameters and equations together with causal and hidden states at all levels. We take 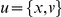 to be the set of all hidden states 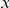 and causal states 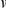 at all levels of hierarchy. The task for the agent is to infer the states 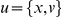 from the sensory input under model *m*. We assume that the parameters (such as 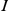, 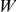 and 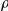, see Figure 4) have been learned previously by the listening bird and are fixed (Table 1).

Our goal is to approximate the *posterior density* 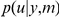 which will give us both the posterior mean of the dynamical states and the uncertainty about this mean. To get a good approximation for the posterior density, we follow a rather indirect way using the marginal likelihood.

The marginal likelihood of 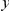 can be written as 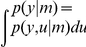. Here, 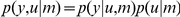 is defined in terms of the likelihood 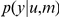 and the prior 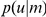. Except for a few analytical cases, this integral is usually intractable and needs to be approximated. One way for this approximation is to introduce a *free-energy* term which is a lower bound for the marginal likelihood. It is not hard to show that:
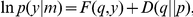
where 
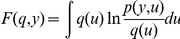
 is the free-energy, 
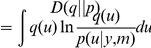
 is the Kullback-Leibler divergence and 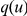 is the *recognition density*. Note that 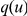 is an auxiliary function that we will use to approximate the posterior density. It is easy to show that 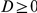, and 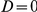 if and only if 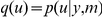. This means 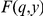 is a lower bound for 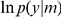, and if we can maximize 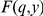, this will minimize 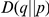 giving an approximation 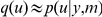 for the posterior density.

To maximize 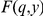 with respect to 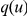, we make the assumption of normally distributed error terms and write 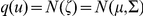 where 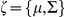 consists of the mode 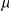 and the variance 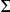. Then the problem turns to a maximization problem of the free energy with respect to 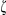:
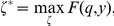
which gives the approximation for the posterior density 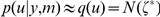. For the details of this variational process and its extension to time-dependent states, see [11].

Since we apply the variational scheme in a hierarchical setting, we write the equations in our model (see Figure 4) in a generic hierarchical form [11]. We use the same set of equations as in the generative model since we assumed the singing and listening birds have the same internal models. We denote *all* hidden and causal states at level 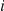 by 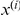 and 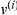, respectively. In particular, 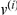 stands for all the 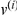 and 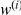 outputs of the 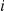 th level. We also write 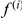 and 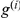 to describe the dynamics of the hidden and causal states in the 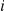 th level:
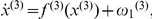


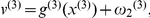


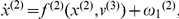


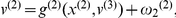


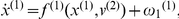


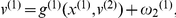
where 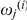 denotes the normally distributed fluctuations at the 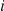 th level. The present model shown in Figure 4 follows this generic form. The causal states (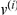) provide input to the subordinate level while the hidden states (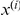) are intrinsic to each level.

Note that the Gaussian fluctuations 
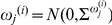
 in the above hierarchical form quantify different amounts of noise at each level of the singing bird. We list the covariance matrices used in the “Ideal Communication” simulation in Table 1. Note that sensory input 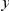 enters the recognition system at the first level: 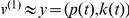. The optimization process of 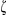 (i.e. the estimated mode of causal and hidden states) can be implemented in a message passing scheme [11] which involves passing predictions down and passing prediction errors up from one level to another. Prediction errors can be written as
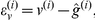


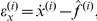
where 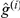 and 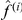 denote the predictions from level above for 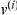 and 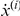, respectively. In this scheme, 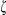 is optimized through gradient descent on prediction errors at each level of the hierarchy. Importantly, the computations required for this gradient descent could be implemented by interacting neuronal populations at each level: Each population comprises causal and hidden *state-units* that encode the expected states and the *error-units*, with one matching error-unit for each state-unit, which encode the prediction errors. The estimated mode of the states, i.e. 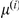, is described by the activity of the state-units. The error units compare the estimated modes with predictions sent via backward and lateral connections and compute prediction errors, which are passed on via forward and lateral connections. This message passing has been shown to minimize precision-weighted prediction errors and optimize predictions at all levels efficiently (see [47], [52] for further details).

*Software Note:* The routines (including commented Matlab source code) implementing this dynamic inversion, which were also used for the simulations in this paper, are available as academic freeware (Statistical Parametric Mapping package (SPM8) from http://www.fil.ion.ucl.ac.uk/spm/; Dynamic Expectation Maximization (DEM) Toolbox).

## Results

To illustrate the behavior of the described generation and recognition schemes, we exposed our recognition model to four different tasks. Since the neuronal structures for song generation and song recognition are mostly different (see Discussion), we refer in the following to the levels in both the generation and recognition models as the first, second and third levels instead of ‘Oscillator’, ‘RA’ and ‘HVC’ levels, respectively.

We first show the case of ‘ideal communication’, i.e. the recognition scheme described above can appropriately infer about the states at all three levels from sensory input that describes a veridical song. In a second simulation, we show the case when the sensory input is not as expected, i.e. when, for the listening bird, there is an unexpected deviation in the song (a single syllable). We will demonstrate how the listening bird detects this deviation and what neuronal correlates are observed in presence of this deviation. In the third simulation, we show that the recognition mechanism is robust against differences in the anatomical connectivity pattern in the second layer. This robustness is a consequence of the hierarchical setup of the generative model. This is an important finding because it explains how different birds can decode the same song although their individual anatomical connectivity within some layers may differ. In our final simulation, we replicate the experimental findings of a study [53] where the authors cooled HVC and observed that the song slowed down. We also show how the listening bird (e.g., female bird in a social context) can detect the minor deviations due to a speed change of the song.

### Ideal Communication

Here, we simulate the ideal situation in which both the ‘singing bird’ and the ‘listening bird’ have learned how exactly a song should sound. As before, we use eight third level ensembles that are each activated sequentially and, during this time, they control the activities of five second level ensembles (Figure 2). The third level imposes a sequence of attractors on the second level which in turn produce linear combinations of appropriate sine waves to produce the air sac pressure and labia stiffness, see Figure 4. To introduce noise (both internal state noise for the singing bird, and also transmission noise to the listening bird), we used normally distributed zero-mean noise with standard deviation of 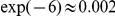 and 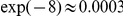 at all levels. To show that recognition is robust against starting condition (i.e. the state of the ongoing neuronal activity within the bird brain at song onset), the initial states of the recognition are chosen differently from the true initial values used in the generation. As expected, we find that the listening bird starts tracking the sensory input very quickly and follows it robustly during the remainder of the song, see Figure 7.

### Deviation from Expected Song

**Figure 8:**
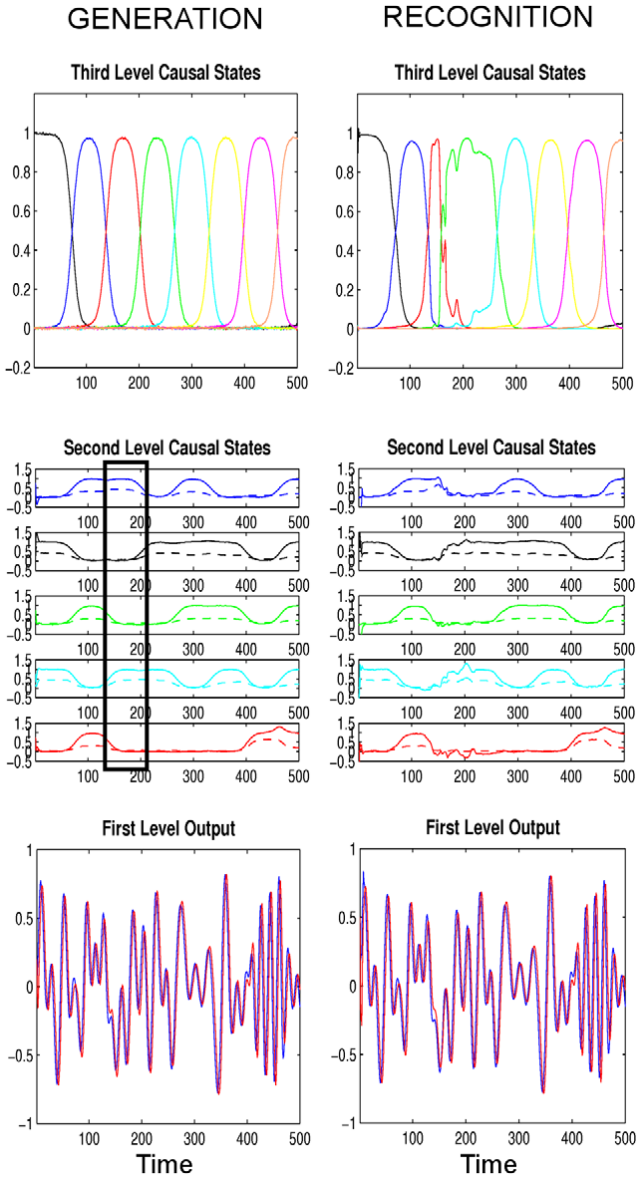
Second simulation ‘Deviation from expected song’: For simplicity, we only show the causal states of the generation (left column) and recognition (right column), where the format is the same as in **Figure 5** with arbitrary units. The listening bird (recognition) hears a slightly deviating syllable between the time steps 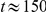 and 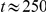 indicated by the black rectangle. During this period, the third ensemble of the HVC level (red color) in the singing bird (generation) activates the first and the fourth ensembles of the RA level (blue and cyan colors) while the listening bird expects the activation of the first ensemble of the second level (blue) only. This unexpected sensory input continues until the listening bird starts hearing and recognizing the expected syllables again after 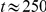. See Figure 9 for plots of the associated prediction errors.

**Figure 9:**
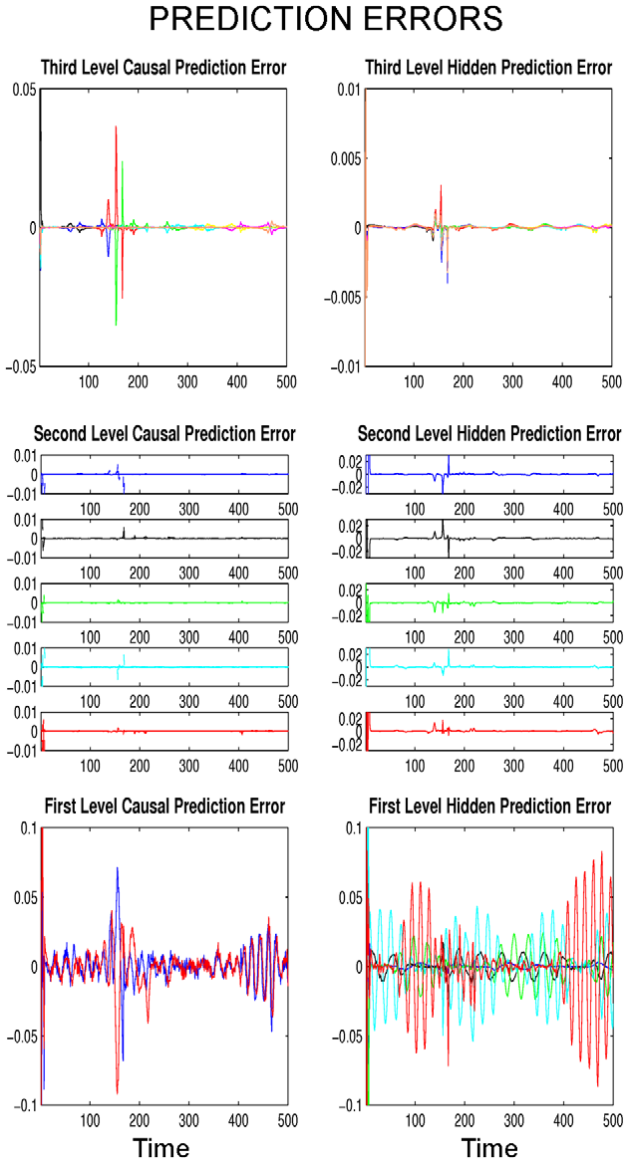
Prediction errors for the ‘Deviation from expected song’ simulation with arbitrary units. Prediction errors for all casual and hidden states during recognition are plotted using the same format as in Figure 5. Note that prediction errors increase during the unknown syllable (between 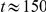 and 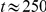) and are observed at all levels.

Next, we show what happens if the listening bird has a different expectation than the singing bird about how a song should sound. In the generative model (singing bird), we use the same third level ensembles and the corresponding second level combinations that we used in the ‘Ideal Communication’ case (Figure 2). However, the recognition system (listening bird) knows a slightly different song where there is a deviation in a single syllable. We model this by changing the effect of the third ensemble at the third level such that it activates only the first ensemble at the second level (instead of the first and fourth as in the singing bird). This means that the motor output and the sonogram look different from the prior expectation of the listening bird but only for the third syllable, see Figure 8. The internal recognition dynamics of the listening bird register this deviation and show two effects during the third syllable, between time points 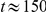 and 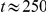: (i) Prediction errors in the recognition are distributed throughout all three levels and are not only explained by changes at a single level (Figure 9). This makes sense since the observed deviation at the first level cannot be explained by the simple oscillatory first level dynamics. Rather, the recognition attempts to explain away the deviation at the first level by using prediction error at the second and third level as well. At the first level, this is quite successful because the recognized dynamics look very similar to the generated dynamics (see Figure 8, bottom row). However, at higher levels, there are obvious differences between the generated and recognized dynamics, i.e. the listening bird can infer a deviation via the prediction error at the second and third levels. (ii) When the deviation has finished, the recognition quickly locks back onto the ongoing song dynamics at all three levels and decodes the song veridically. In summary, this simulation shows that the dynamic recognition hierarchy uses all its levels to compensate for unexpected deviations in the song. This means that all levels of the hierarchy work together in concert to minimize the effects of deviations throughout the hierarchy. In other words, the activity of high-level auditory processing levels in songbirds in response to small deviations in the expected song may be most revealing for their function. This mechanism may be important in social context since the listening bird can recognize subtle variations in the singing bird by its activity in high-level areas and grade the singing bird's overall performance [54].

### Differently Wired Brains: Communication within Species

Considering the anatomical complexity of the brain, genetic and developmental variability is expected in the brains of individuals of the same species. At the macro scale, the general connectivity structure of distinct brain regions may be shared, but at the micro scale, variability is found in size, location and connections between individual neurons or neuronal ensembles [55]–[58]. Here, we simulate a difference in the connectivity structures by using different second-level connectivity matrices *W* (Figure 4 and Eq. (3)) in the generative model of the singing bird and the recognition system of the listening bird. In other words, the listening bird has a different internal model at the second level as would be prescribed by the generative model of the singing bird at the RA level. How can birds with individual variability in their internal models still extract the same information from a song?

**Figure 10:**
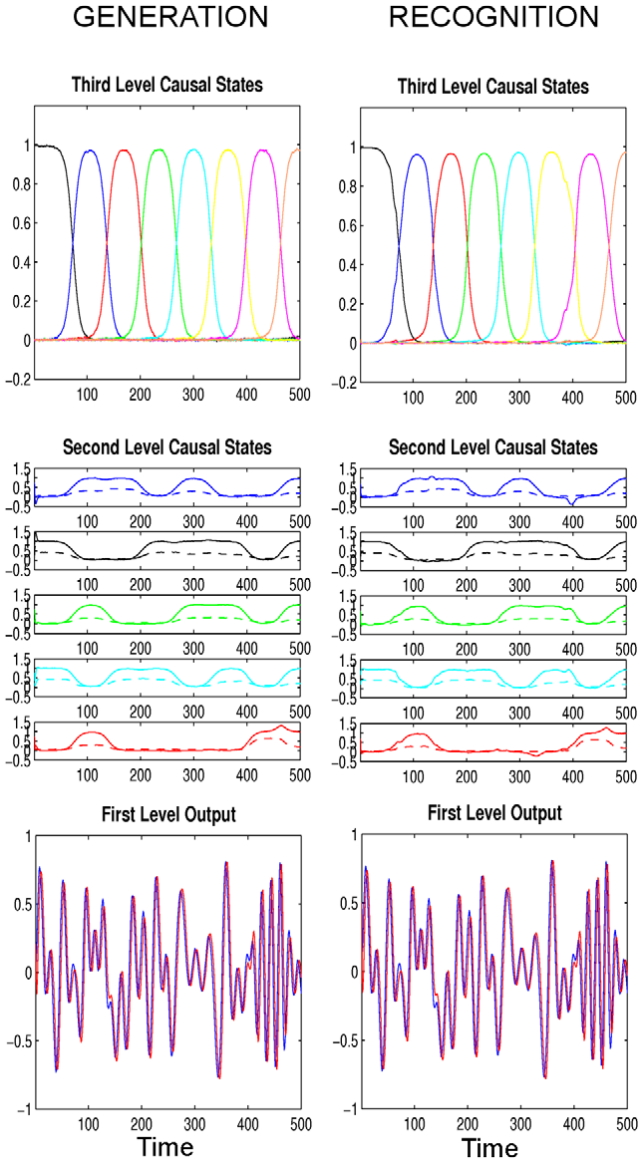
Third simulation ‘Differently wired brains’: For simplicity, we only show the causal states of the generation (left column) and recognition (right column), where the format is the same as in **Figure 5** with arbitrary units. The connectivity matrices (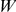
's) at the second level are different in the singing bird (generation) and in the listening bird (recognition). Recognition still works as well as in the first simulation (‘Ideal communication’, Figure 7) because third level ensembles can compensate for this variability by providing different input to the second level (different *I* vectors).

The answer is that differences in the second-level connectivity matrix *W* can be compensated by a different driving activity *I* from the third level since *I* depends on *W* (see Theorem 1 in Text S1). In our simulation, we assume that these driving activities have already been learned in the corresponding birds, e.g. during juvenility. As shown in Figure 10, the states at all three levels can be recognized successfully even though the second levels in the two birds are wired differently. This means that the internal models of generation and recognition do not have to be the same but can cope with structural variations due to anatomical variability at the micro-scale. Critically, this compensation of anatomical variability at the second level relies on the hierarchical configuration and learning of the connectivity from the third level to second level.

### Cooling of HVC

In song generation, a critical question is which regions of the brain are involved in the timing of syllables or sub-syllable structures. A recent study tackled this question by manipulating the temperature of the HVC and RA regions in the singing bird [53]. Importantly, it was shown that song speed at all time scales slowed down but the acoustic structure stayed the same as the temperature of HVC dropped. In the sonogram, this corresponds to a temporal stretching of the song. Conversely, cooling of RA did not have any effect on the timing of the song. This suggests that HVC is involved in the control of the timing of the song [53].

**Figure 11:**
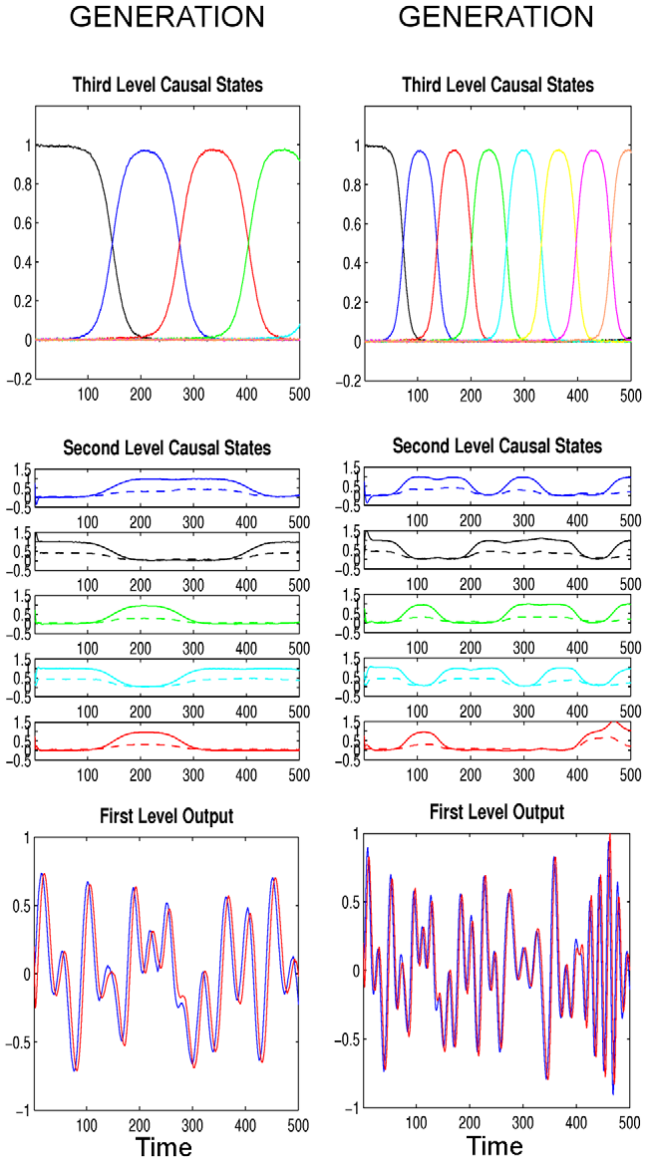
Generated dynamics for the fourth simulation ‘Cooling of HVC’: We simulated two cooling experiments, where the format is the same as in **Figure 5** with arbitrary units. Left: The rate constant at the HVC (third) level, 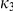, is decreased by half. Right: The rate constant of the RA level (second level), 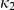, is decreased by half. The change in 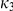 slows down the dynamics of the system, while cooling at the RA level does not have any significant effect, compare with the dynamics in Figure 5. The parameters used are 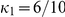, 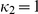 and 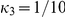 on the left and 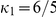, 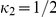 and 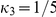 on the right.

We observed similar behavior in our model where we modeled the cooling by manipulating the rate (i.e. speed) constants 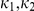 and 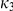 at the three levels. Importantly, changing the rate constant for HVC slows down the song but changing the rate constant for RA does not. In the first simulation (Figure 11, left), we ‘cooled’ HVC by changing 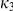 from 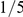 to 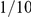. This slows down the dynamics of the HVC level and immediately slows down the RA level as well since the control signals coming from HVC now last twice as long. In other words, we find as in the cooling experiment that HVC, due to its position at the top of the hierarchy, controls directly the timing of the song. To reflect this slowing down in the output we also changed 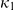 from 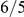 to 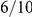 (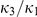 is kept constant in all simulations) to adjust the frequencies which were chosen independently from the RA level for simplicity (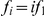 where 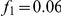). In the second simulation (Figure 11, right), we changed the rate constant of RA, 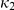, from 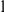 to 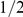. This has no observable effect, as in the experiment [53], on the dynamics of RA ensembles since the timing of attractor activations is controlled by the timing of HVC. A change in 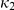 only slows down the transition times which has no detectable effect in the output.

**Figure 12:**
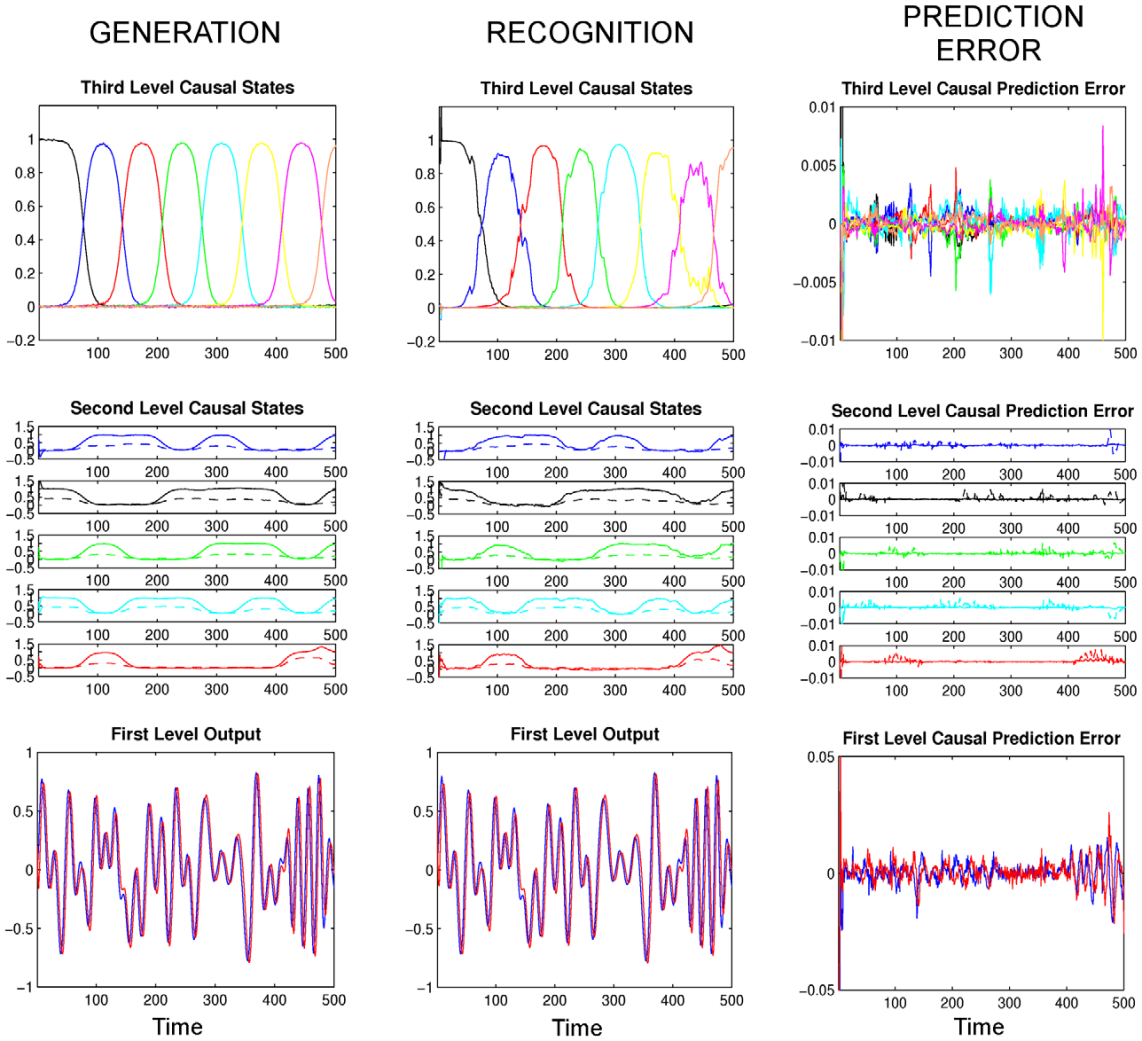
Recognition results for the fourth simulation ‘Cooling of HVC’, where the format is the same as in **Figure 5** with arbitrary units. Left: We slowed down the singing bird by decreasing the rate constants by 3%: 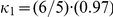, 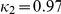 and 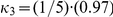. Middle: The rate constants for the recognition are 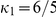, 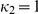 and 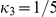. Right: The listening bird can distinguish this subtle change in song speed as can be seen from the prediction errors of the causal states at all three levels. (The hidden states show similar prediction errors at all levels).

Speed changes may not only have an experimentally observable effect in the generated song but also in the listening bird. Interestingly, speech changes in song also occur under natural conditions, e.g. in a social context: Male birds sing slightly faster when addressing a female bird (directed song) compared to singing towards other males or when alone (undirected song) [6], [59]. Using the present model, we tested whether the listening bird can detect such small changes in the singing bird during directed song. We slowed down the song by 3%, thereby modeling an undirected song, and analyzed the prediction errors in the listening bird which expected the slightly faster, directed version. The listening bird was able to recognize the song successfully but it also reliably distinguished the subtle change in the tempo, as can be seen from the sustained prediction errors at all three levels (Figure 12).

## Discussion

We have described a hierarchical model for generating birdsongs and introduced an online Bayesian inversion as a recognition model. The key result is that the specific anatomical, functional and hierarchical structure of birdsong generation enables Bayesian online decoding of hidden information at a slow time-scale at the HVC and RA levels. Four simulations showed that the Bayesian recognition mechanism works efficiently in several settings and its functional behavior might be helpful to understand the mechanisms of birdsong recognition. In addition, recognition is robust to noise and can be performed online. Overall, this is a unified modeling approach which handles both generation and recognition of birdsong and may serve as a model for vocal bird communication.

Both generation and recognition models extend previous modeling work either by using novel techniques (e.g. Bayesian inference for hierarchical, stochastic, nonlinear dynamical systems) or by combining well-known nonlinear differential equation systems in a novel way (generative model). The model explains recognition of birdsong as continuous message passing scheme among auditory areas and explains the dynamic song recognition system of birds using Bayesian techniques. In the generation model, we combine a well-established syringeal model with the sequential HVC/RA model and describe a hierarchical and dynamical mechanism which transforms the spatiotemporal coding at the RA level into the rich, complex structure of the song power spectrum. Based on this generative model, we use Bayesian inference to model song recognition by a conspecific. This modeling strategy is a novel approach to employ experimental findings in birdsong generation for establishing a functional model of birdsong recognition. In fact, decoding of sensory input generated by hierarchical, nonlinear dynamical systems is usually technically challenging and often impossible [60], [61] because the sensory input may not be informative about hidden information at higher levels. However, here we found that the decoding of birdsong using hierarchical Bayesian inference based on a song generation model is feasible, robust and can be performed online. Intuitively, it may be obvious that birdsong must be generated such that conspecifics can derive information (meaning) from it. The question is how birds do this mechanistically. Here, we propose that this recognition mechanism may rest on Bayes-optimal inference given the specific hierarchical arrangement of the neuronal birdsong-generating network.

### Neurobiological Plausibility of the Recognition Model

We have derived a recognition scheme using Bayesian inference. However, bird brains may have established their recognition capabilities by evolutionary processes [4], [49], [62]. What are the similarities between the proposed recognition scheme and the biological one?

Note that the present modeling does not suggest that the areas involved in generation and recognition are the same. Many computations during recognition are different from those in generation. The present recognition scheme consists of three hierarchical levels, thereby mirroring the hierarchical generation system. We found that three hierarchical levels are also appropriate for the recognition of a song. Interestingly, experimental findings point to a hierarchical arrangement of the auditory system in songbirds as three major functional levels of processing [63], [64] where it is partially unclear yet how this hierarchy maps exactly onto the auditory system. Moreover, note that these areas are mostly investigated for male (zebra finch) birds and it is quite possible that there could be different areas involved in females or in other bird species.

Experimental evidence suggests that HVC may be located at the highest level of this recognition system. In particular, HVC_(X)_ neurons (HVC neurons that project to Area X, see Figure 1) are selectively responsive to the bird's own or a conspecific's song [65], [66]. The firing of HVC_(X)_ neurons at temporally precise times during an auditory stimulus [65] is similar to the temporally precise activation of HVC_(RA)_ neurons during singing. This suggests that HVC_(X)_ neurons may be involved in the representation of the expected sequence of song dynamics. In the present model, the third level encodes both the sequence prediction but also the perceived deviation from this sequence.

The circuitry of areas subordinate to HVC during song recognition is not particularly well understood. The caudal mesopallium (CM) and caudomedial nidopallium (NCM) have been shown to be selective for particular familiar songs or sounds and are involved in auditory memory [23], [63], [64]. Similar functions are implemented by the second level of the present recognition model: The second level encodes the expectation of specific spatiotemporal patterns, i.e. it encodes auditory memory by attractors that correspond to specific vocal tract dynamics (sounds). Note that there is a clear distinction between the third and second level in the model: While the third level encodes the expected sequence of sound dynamics, the second level encodes the repertoire of song sounds (transcribed to sound waves by the vocal tract dynamics). This functional separation is also assumed to be implemented in the real bird brain [26].

In the primary auditory area, Field L, spectral-temporal receptive fields (STRF) have been proposed to explain the selective responses of neurons [67]. These selective responses may correspond to the recognition dynamics at the first level in the model which decodes the detailed spatiotemporal structure of the auditory stimulus guided by higher level predictions. It is interesting to note that we could use the present recognition model to derive, as done experimentally [67], the spectral-temporal receptive fields at the first level. Alternatively, one could use experimentally acquired STRFs to adapt the first level of the present model to establish exact equivalence of the model and the real system at the level of primary auditory areas.

### Relation to Other Generation Models

There are several models that focus on the sequential activation of HVC_(RA)_ neurons using single neuron models. Inhibition is believed to be a key element to generate rhythmic (sequential) activity in HVC [15], [16], [68]. We used winnerless competition which relies on inhibition to sequentially activate HVC_(RA)_ ensembles. A similar generation mechanism as described here can be obtained using the synaptic chain scheme: Li and Greenside [12] proposed a conductance-based model for HVC_(RA)_ neurons from which they obtained sequential multi-spike bursts. Later, Jin et al. [13] used an intrinsic bursting mechanism to obtain higher firing rates more consistent with the experimental data. This scheme was extended in [14] and was shown to produce robust and highly stereotyped sequential bursts. A learning mechanism was proposed in [69] showing how a sparse temporal code can emerge from a recurrent network. The models mentioned above focus on describing possible ways for the sequential activity of HVC where the downstream areas can be regarded as driven in a feed-forward fashion by HVC. A comprehensive generative model that includes HVC, RA and motor control areas was described in [18]. This study showed that the intrinsic connectivity at the RA level can substantially influence the acoustic features of syllables. This approach is similar to the present where the common research question is which parameterization (connectivity) of a recurrent neural network will generate motor control signals that result in realistic acoustic features of birdsong. However, we additionally incorporated recent findings [26] which point to a specific role of RA ensembles in encoding sound wave modulations. Furthermore, we provide evidence that the hierarchical setting of HVC and RA ensembles is the basis for robust and rapid song recognition.

### Relation to Other Recognition Models

Theunissen et al. [70] estimated spectral-temporal receptive fields (STRF) of nonlinear auditory neurons using natural sounds as sensory input. The STRFs describe which temporal succession of acoustical features would elicit the maximal neural response and provide useful information for modeling perception of acoustic features, e.g. in the primary auditory area, Field L [67]. A two-level model was introduced [71] where the first level encoded frequency responses identified by an STRF analysis and the second level used these features to model song selective responses of HVC neurons. In another approach, Larson et al. [72] proposed a model for auditory object recognition where the first level uses a distance metric to distinguish between different spike trains and the second level acts as a decision network. However, both of these models propagate auditory signals in a feed-forward fashion from the low to the high level while the present scheme uses dynamical and recurrent bottom-up and top-down message passing thereby providing a more comprehensive model of the neuronal dynamics observed during song recognition.

Learning models such as [73] and [74] were proposed which also include birdsong production and evaluation. These models mainly focus on the neural mechanisms of learning but they also provide mechanisms for song evaluation.

There have been also attempts for the automated recognition of birdsongs using machine learning methods, e.g. [75], [76]. However, these models are not concerned with neurobiological plausibility but rather use ad-hoc techniques as used in automated speech recognition, i.e. hidden Markov models and template-based matching of song syllables.

### Implications for Empirical Research

There are several implications for future experiments which one can derive from the present model. The first is that we observe prediction errors at all levels when there is an unexpected piece of song (Figure 9) or a song which is slower than expected (Figure 12). This suggests that there may not be a single area in the auditory pathway (such as HVC_(X)_ or LMAN in the anterior forebrain pathway) that acts as a comparator between the stimulus and previously memorized tutor song [77] but several levels of the auditory pathway may be involved in this comparison. Comparing the neuronal recordings from a bird that listens to a normal speed song and a slower version of the same song might reveal the locations where these prediction errors are computed. Similar experiments have been done in auditory areas Field L and caudal lateral mesopallium (CLM) where some neurons responded robustly to perturbations in vocalization or playback of the bird's own song [21]. A functional model like the one presented here could predict what amount of activity should be expected in experiments given defined deviations, at different levels of the recognition hierarchy. Parallel to this idea, a recent experiment explained the activity in CLM by the level of *surprise* in the stimulus [20]. Our model could be used to predict the amount of surprise or prediction error at different hierarchical levels. As the present model covers much of the auditory pathway, this prediction technique may be best suited for using functional MRI on birds [78], [79] where one would model increased activation, relative to some baseline condition, as an increase in prediction error.

### Potential Relationship between Birdsong and Human Speech

As noted by several authors, human speech and birdsong have in common that both are complex, hierarchical, sequenced vocalizations which are repetitions and combinations of simple units such as phonemes and syllables [2], [80], [81]. Although human speech is far more complex than birdsong, the underlying anatomical and functional features show striking similarities such as the pathways for vocal production, auditory processing and learning [22], [81]. Songbirds, similar to humans, gain their vocal abilities early in life by listening to adults, memorizing, and practicing their songs [22]. These similarities suggest that one may derive insight about human speech recognition and learning from findings in birdsong research [82].

The present results clearly point to the usefulness of a hierarchical recognition structure to decode sequences of syllables. Such hierarchical models are rarely used in automated speech recognition [83] presumably because the standard model, the hidden Markov model, is mathematically best understood only in a non-hierarchical setting. The present scheme shows that complex spectral dynamics such as birdsong may be modeled as a sequence of nonlinear dynamics, where, in the generative model, each level drives the subordinate level in a highly non-linear fashion. To invert such a hierarchical, nonlinear, dynamical system, one requires sophisticated Bayesian inference machinery [11], [84]. We described such a mechanism previously for a simple auditory sequence of sounds [85]. The novelty of the current approach is that we use a neurobiologically plausible generative model to derive a functional recognition model that has also the potential to recognize real and complex birdsong. In addition, we hypothesize that the specific arrangement of HVC and RA level (dynamic sequences driving attractor dynamics at a lower level) and its Bayesian online inversion will not only play a role in birdsong recognition models but may be successfully used for automated speech recognition as well.

### Further Extensions to the Model, Scaling and Sensitivity Analysis

The mathematical model that we used to generate birdsongs was previously shown to produce accurate copies of songs such as canary [30], chingolo sparrow [17], white-crowned sparrow [31] and cardinal [45] songs. The vocal organs of other birds, e.g. of the zebra finch, can generate highly nonlinear, more complex, acoustic dynamics than the one considered here. For modeling such songs, one would have to replace the syrinx model of Eq. (4) by a more involved syrinx model such as the one reported in [86].

For our purposes, we focused on one particular song to describe the generation and recognition framework. The recognition of different songs either by the same or different conspecifics could be modeled by using multiple sequences encoded at the third level, where we assume that the recognition will converge to the best fitting sequence. In addition, one could adapt the nonlinear syrinx model to endow a singing bird with its own low-level acoustic characteristics.

In the present model, we used rather small numbers of ensembles for visualization and computational purposes. The generative model applies to an arbitrary number of ensembles and similar type of dynamics can be obtained with larger number of ensembles at each level (see Figure S1 for generation with 100 HVC ensembles). For recognition, we performed similar experiments with larger numbers of HVC ensembles (32) and RA patterns (24) where the recognition results were as robust as with the reported smaller size models (see Figure S2 for the simulation). This indicates that the model scales to larger model sizes. However, there are two main issues that one will need to address to enable recognition using hundreds of units: (i) The computational power required for the recognition quickly increases with the number of ensembles used (with complexity 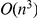 due to computing a matrix exponential, see [11]). This can be resolved by parallelizing the ensemble-specific computations which would be a further step towards biological reality. Currently, we emulate these parallel computations using a single-process Matlab implementation. (ii) The complexity of the syrinx model must be matched by the ‘descriptive power’ of the RA level. In other words, if one wanted to increase the number of RA ensembles significantly, one also had to render the model at the syrinx level more complex so that the recognition can infer more RA ensembles from more complex sensory data. However, this increase in model complexity at the syrinx and RA levels would require a more sophisticated syrinx model and is beyond the scope of the present work, in which we provide a proof of concept and introduce the computational framework.

Furthermore, we tested the sensitivity of the Bayesian recognition in response to changing specific details of the generative model: (i) We used higher noise levels (standard deviation of 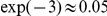 and 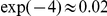) as compared to the simulations above, the recognition still robustly inferred the hidden states and causes at all levels (see Figure S3) (ii) We found that the recognition is robust against varying the initial conditions of the states in both the generative model and recognition. We tested a wide range of random initial conditions in both generation and recognition and observed that in all simulations the recognition quickly locks into the necessary dynamics. This implies that the listening bird can recognize a song reliably whatever the initial state of itself or the singing bird at the beginning of the song. (iii) We also changed the connectivity matrices at the third level (with the constraint of high inhibition from the previous neuron and low inhibition to the next neuron) and at the second level (with the constraint that global stability conditions are satisfied, see Theorem 1 in Text S1) of the generative and recognition models. The recognition was still robust with these different connectivity matrices (see Text S1 and Figure S4).

### Conclusion

We described a model to generate artificial birdsongs and a scheme for their online recognition. We constructed a model based on key experimental findings in birdsong generation. Our results show that the specific, hierarchical mechanism how birdsong is generated enables robust and rapid decoding by a hierarchical and dynamic Bayesian inference scheme. We have interpreted this as evidence that the birdsong generation mechanism is geared toward making the song robustly decodable by conspecifics and discussed the experimental evidence that songbirds use a recognition mechanism similar to the present Bayesian inference scheme.

## Supporting Information

Audio S1 Sound file for the generated artificial birdsong obtained by plugging the first level output of the generation scheme (Figure 5C) into the syrinx equations, Eq. (4).
(WAV)

Figure S1 Generated dynamics with 100 HVC ensembles at the third level where the format is the same as in Figure 5 with arbitrary units. We only modified the rate constants (so that all activations fit to the time-window used) of the generative model and the rest of the constants are the same and listed in Table 1. This simulation shows that the generative model can be scaled up and similar dynamics as shown in the main text figures can be obtained with long HVC sequences.
(TIFF)

Figure S2 The dynamics of song generation (left column) and song recognition (right column) with 32 neuronal ensembles at the third levels of both generation and recognition models. The format is the same as shown in Figure 5 with arbitrary units. We only modified the rate constants (so that all activations fit to the time-window used) and the rest of the constants are the same and listed in Table 1. This simulation shows that the recognition model can be scaled up and similar recognition dynamics as shown in the main text figures can be obtained with long HVC sequences.
(TIFF)

Figure S3Robustness to noise of both the generative and recognition models: We generated song dynamics (left column) and song recognition (right column) using higher noise levels than in the simulations reported in the main text. The format is the same as shown in Figure 5 with arbitrary units. We used noise with standard deviation of 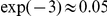 and 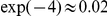 for causal and hidden states, respectively, at all levels of the generative model. The recognition was still robust at these noise levels. For simplicity, we only show the causal states of the generation and recognition.(TIFF)

Figure S4 Robustness of the generative and recognition models with respect to the connectivity matrices at the third and second levels. The format is the same as shown in Figure 5 with arbitrary units. In this simulation, we used different (randomly assigned) connectivity matrices at the third and second levels of the generative and recognition models and obtained qualitatively the same dynamics as in the simulations reported in the main text.
(TIFF)

Text S1 Further details about the RA and oscillation levels and the description of the online Bayesian recognition. We describe how to choose *I* vectors to control the RA dynamics and explain Eq. (5) for the oscillatory dynamics in the first level. In addition, we describe the sensitivity analysis of the model with respect to the changes in the connectivity matrices.
(PDF)
