# Supplementary figures and images for: A Hierarchical Neuronal Model for Generation and Online Recognition of Birdsongs
Source: PLoS Comput Biol. 2011 Dec 15;7(12):e1002303. doi: 10.1371/journal.pcbi.1002303 (PMC3240584; doi:10.1371/journal.pcbi.1002303)

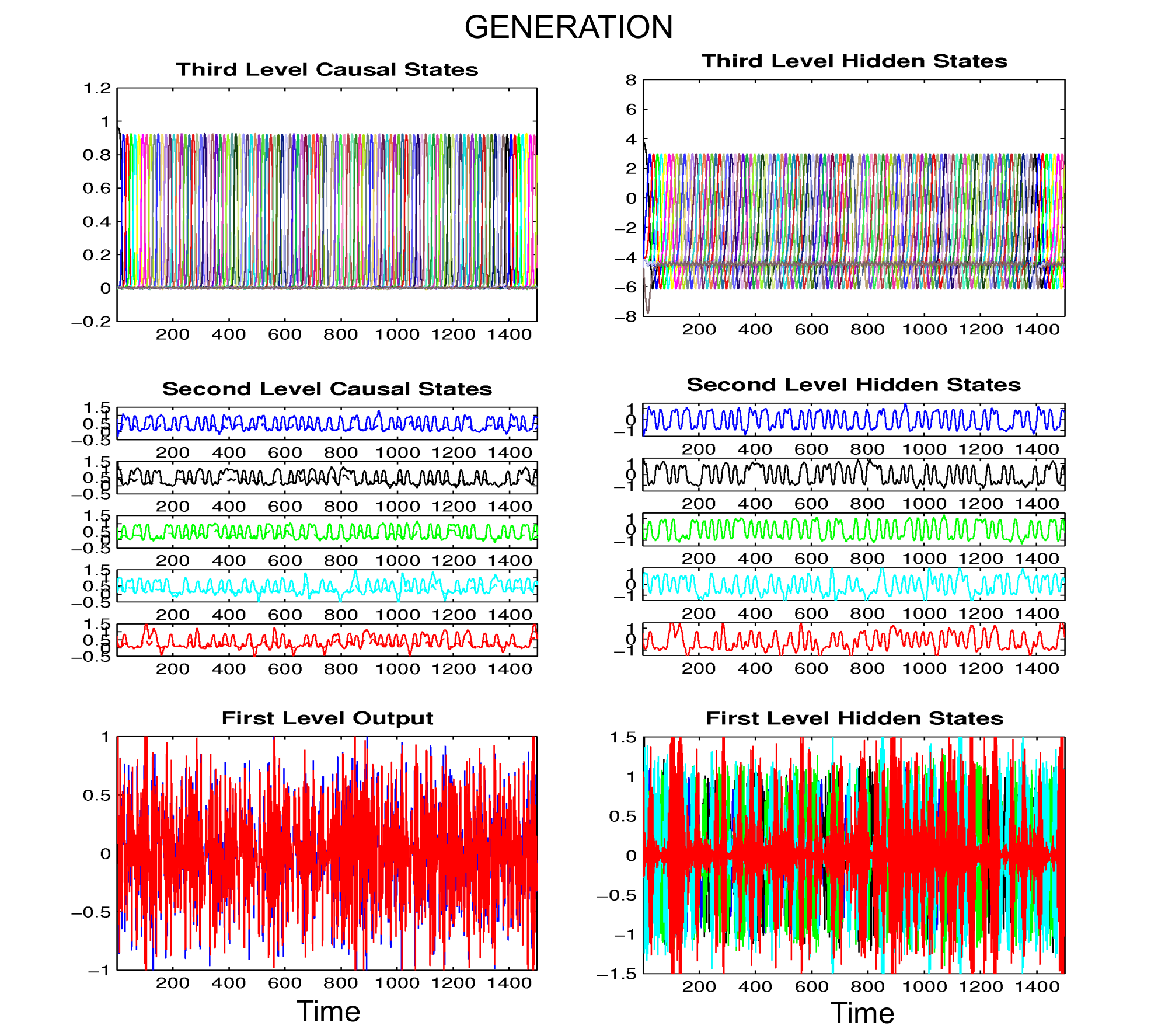

Supplement: Figure S1 — Generated dynamics with 100 HVC ensembles at the third level where the format is the same as in Figure 5 with arbitrary units. We only modified the rate constants (so that all activations fit to the time-window used) of the generative model and the rest of the constants are the same and listed in Table 1. This simulation shows that the generative model can be scaled up and similar dynamics as shown in the main text figures can be obtained with long HVC sequences. (TIFF) [file pcbi.1002303.s002.tiff]

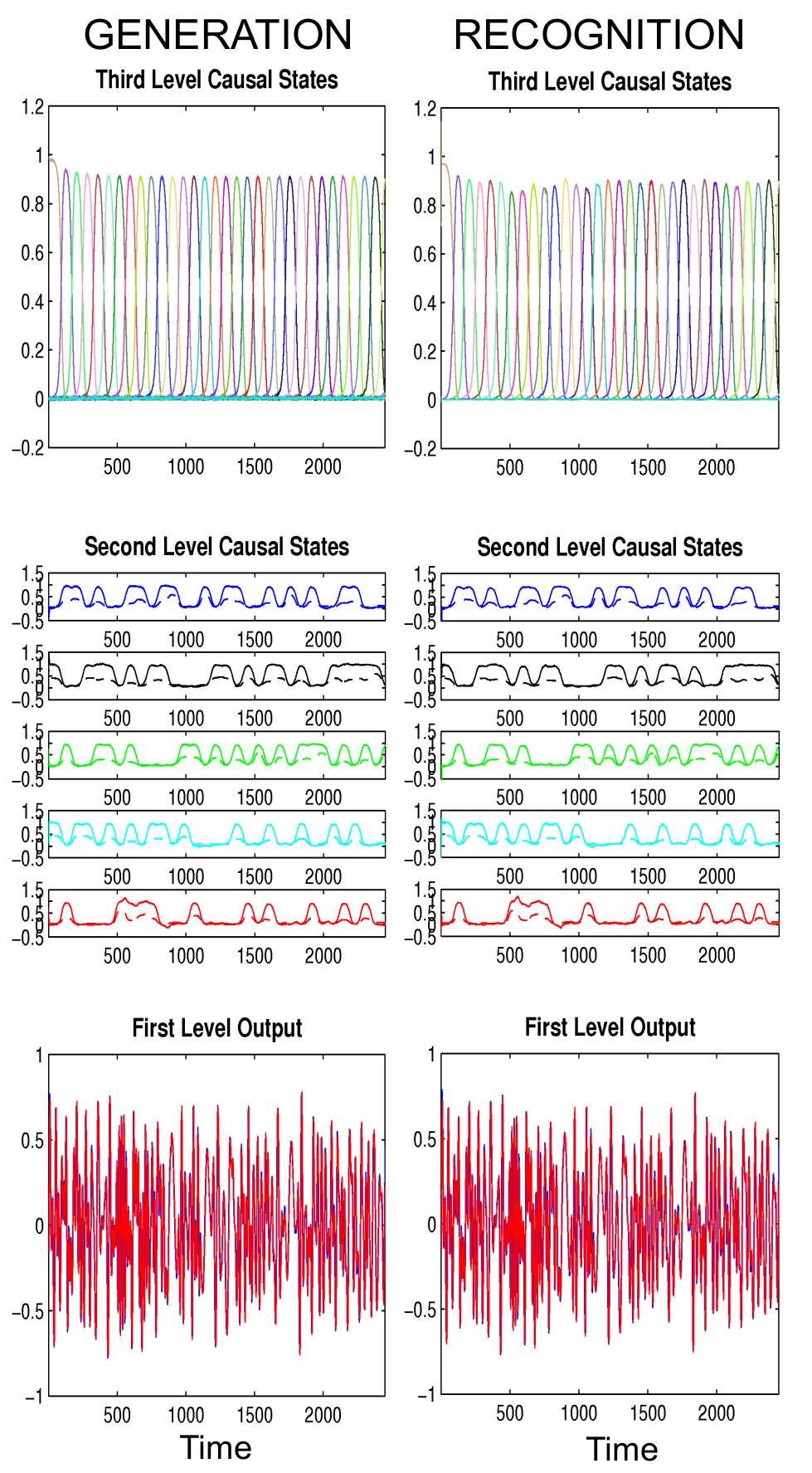

Supplement: Figure S2 — The dynamics of song generation (left column) and song recognition (right column) with 32 neuronal ensembles at the third levels of both generation and recognition models. The format is the same as shown in Figure 5 with arbitrary units. We only modified the rate constants (so that all activations fit to the time-window used) and the rest of the constants are the same and listed in Table 1. This simulation shows that the recognition model can be scaled up and similar recognition dynamics as shown in the main text figures can be obtained with long HVC sequences. (TIFF) [file pcbi.1002303.s003.tiff]

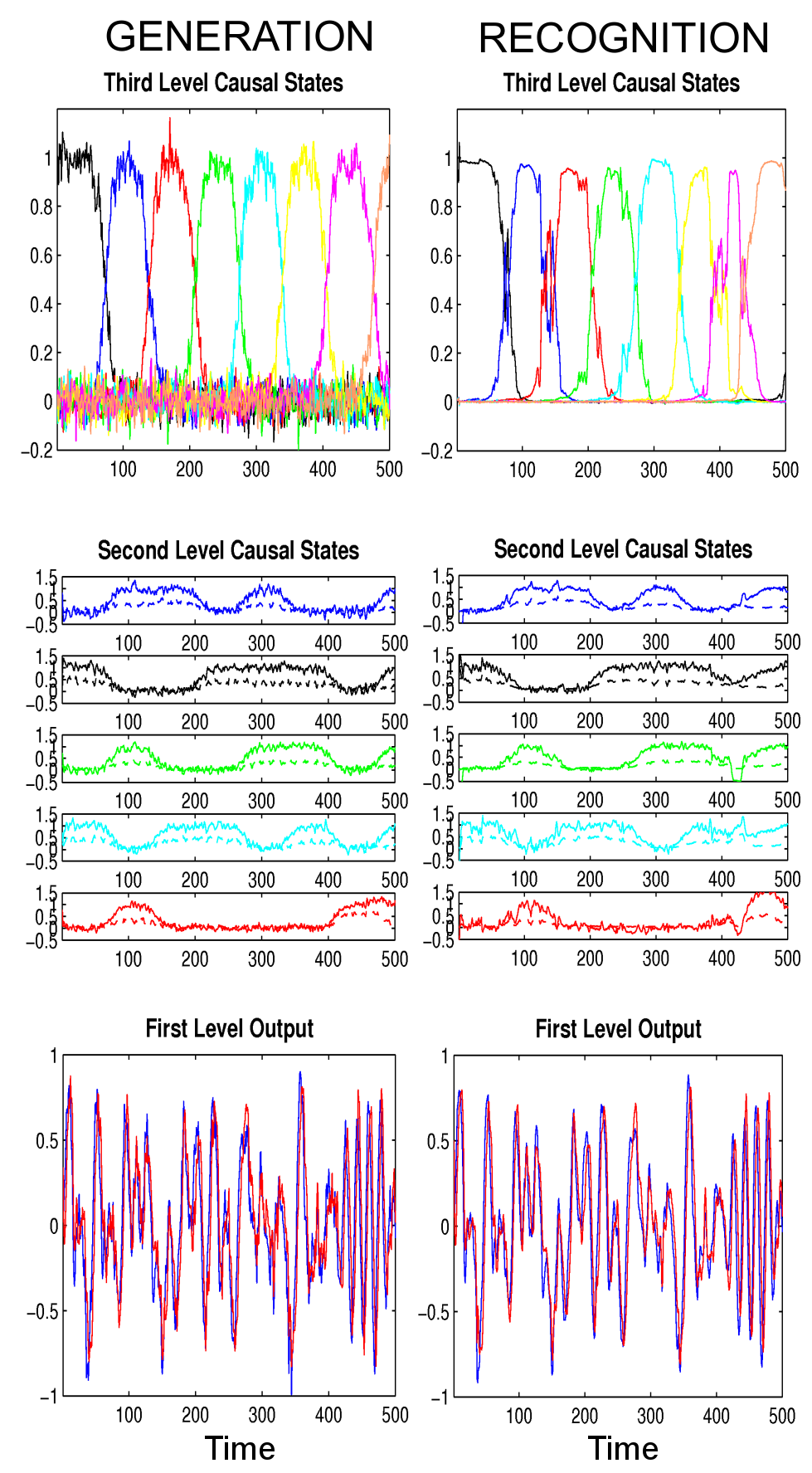

Supplement: Figure S3 — Robustness to noise of both the generative and recognition models: We generated song dynamics (left column) and song recognition (right column) using higher noise levels than in the simulations reported in the main text. The format is the same as shown in Figure 5 with arbitrary units. We used noise with standard deviation of and for causal and hidden states, respectively, at all levels of the generative model. The recognition was still robust at these noise levels. For simplicity, we only show the causal states of the generation and recognition. (TIFF) [file pcbi.1002303.s004.tiff]

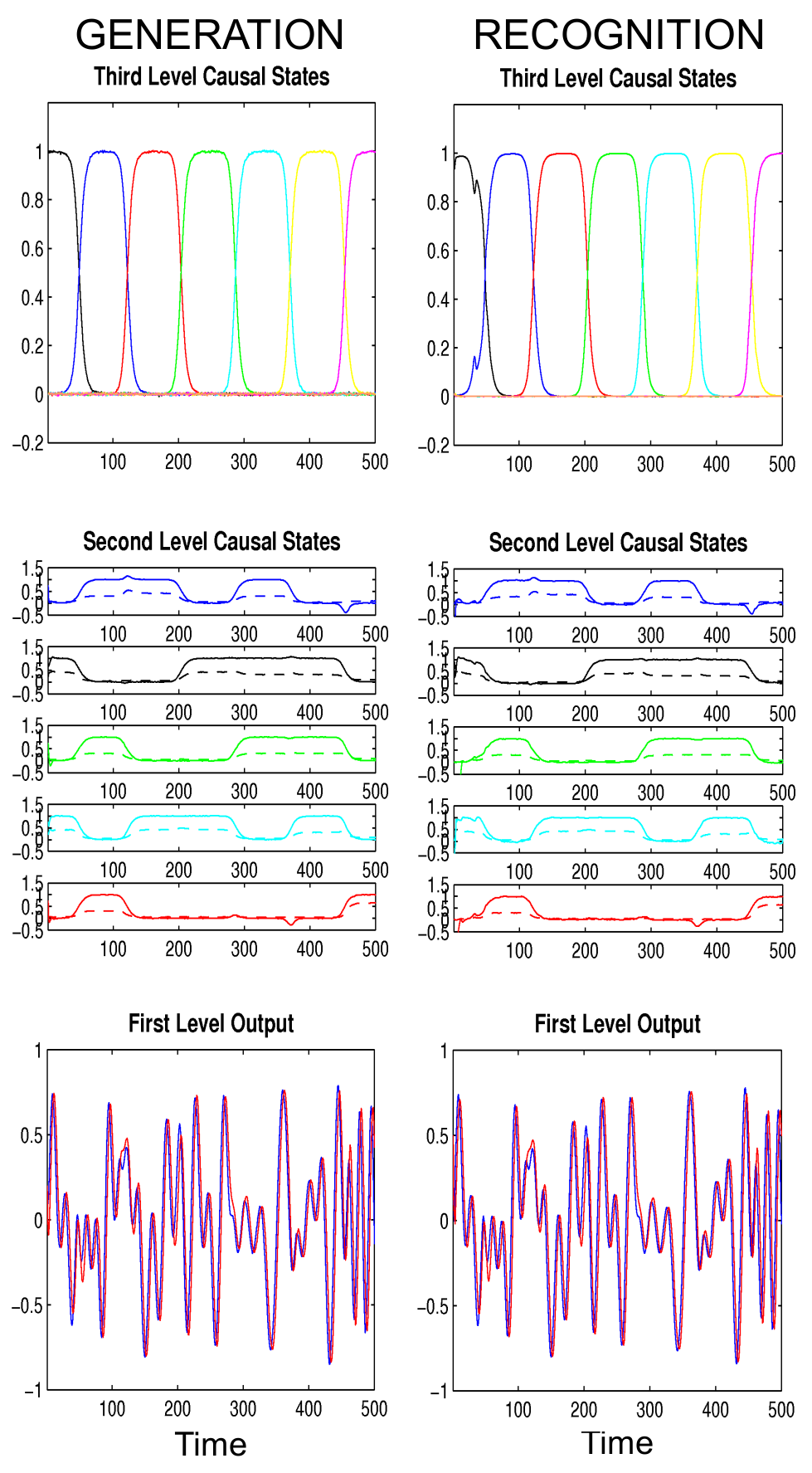

Supplement: Figure S4 — Robustness of the generative and recognition models with respect to the connectivity matrices at the third and second levels. The format is the same as shown in Figure 5 with arbitrary units. In this simulation, we used different (randomly assigned) connectivity matrices at the third and second levels of the generative and recognition models and obtained qualitatively the same dynamics as in the simulations reported in the main text. (TIFF) [file pcbi.1002303.s005.tiff]
