# Supplementary material for: A Hierarchical Neuronal Model for Generation and Online Recognition of Birdsongs
Source: PLoS Comput Biol. 2011 Dec 15;7(12):e1002303. doi: 10.1371/journal.pcbi.1002303 (PMC3240584; doi:10.1371/journal.pcbi.1002303)
Supplement: Text S1 — Further details about the RA and oscillation levels and the description of the online Bayesian recognition. We describe how to choose I vectors to control the RA dynamics and explain Eq. (5) for the oscillatory dynamics in the first level. In addition, we describe the sensitivity analysis of the model with respect to the changes in the connectivity matrices. (PDF) [file pcbi.1002303.s006.pdf]

Supplementary Text S1

RA Dynamics

Under some conditions on W and A, one can obtain the desired attractor in the network given by Eq. (2) by properly choosing *I*. The proof of the following theorem can be found in [1] and [2].

Theorem 1. Let $H = (W + W^T)/2$. If $H - A$ is negative definite then the network given by Eq. (2) has a unique globally asymptotically stable equilibrium point.

Note that we take $A = diag(a)$ to be a diagonal matrix with all the diagonal elements equal to a positive real number a. Entries of W are chosen randomly in $[-1,1]$ with the restriction of $H - A$ being negative definite. Now let's assume x^* is the desired state for which the entries are either 1 or -1. It is not hard to check that if one chooses I to be $I = c\phi(x^*) - W\phi(x^*)$ where $c = a/\tanh(1) = -a/\tanh(-1)$, the vector x^* becomes an equilibrium point of the network, Eq. (2). In this way, we can choose the proper I_k vector for the *k*th HVC ensemble to create the desired attractor in the RA level.

Mixtures of RA-modulated Oscillators at the First Level

Here, we describe how to obtain the hidden states $x_i^{(1)}$, $y_i^{(1)}$ and the outputs $v^{(1)}$, $w^{(1)}$. Note that, to drive the vocal model appropriately, we produce two outputs (the second output is simply a time-shifted copy of the first one), which are used in producing the air sac pressure *p(t)* and the stiffness of the labia *k(t)*. We introduce the following equations, where we use superscripts to denote the vectors at the i th level:

$$\dot{x}_i^{(1)} = v_i^{(2)} \sqrt{1 + (f_i \kappa_1)^2}\, \sin(f_i \kappa_1 t) - x_i^{(1)} + \omega_1^{(1)},$$

$$\dot{y}_i^{(1)} = v_i^{(2)} \sqrt{1 + (f_i \kappa_1)^2}\, \sin(f_i (\kappa_1 t - \theta)) - y_i^{(1)} + \omega_2^{(1)},$$

$$v^{(1)} = \sum_{i=1}^{n} w_i^{(2)} x_i^{(1)} + \omega_3^{(1)}, \qquad (5)$$

$$w^{(1)} = \sum_{i=1}^{n} w_i^{(2)} y_i^{(1)} + \omega_4^{(1)},$$

where $x^{(1)}, y^{(1)} \in \Re^{n\times1}$ are the hidden states of the first level, $v^{(1)}, w^{(1)} \in \Re^{n\times1}$ are the output vectors, $f = (f_i) \in \Re^{n\times1}$ is the vector of angular frequencies, κ_1 is a scalar, $\omega_j^{(1)}$ are normally distributed noise vectors and in the remaining variables, subscripts denote the ith entry of the corresponding vector. The term $\sqrt{1+(f_i\kappa_1)^2}$ acts as a normalizing constant for the solutions.

Assuming that the RA output $v_i^{(2)}$ is constant (by construction RA dynamics are typically close to 0 or 1 except for the transition times), the analytic solution of the first two equations above are $x_i^{(1)} = v_i^{(2)} \sin(f_i\kappa_1 t + \phi_i) + c_i e^{-t}$ and $y_i^{(1)} = v_i^{(2)} \sin(f_i(\kappa_1 t - \theta) + \phi_i) + d_i e^{-t}$ where ϕ_i's are the phase-shifts given by $\phi_i = \arctan(-f_i\kappa_1)$. The constants c_i and d_i depend on the initial conditions. This means that states, regardless of initial conditions, are quickly attracted to the desired sine functions since the term e^{-t} diminishes the effect of the initial conditions quickly. Note that this implies that $y_i^{(1)}$ is just a shifted copy of $x_i^{(1)}$ by θ/κ_1. The output vectors $v^{(1)}$ and $w^{(1)}$ are a linear combination of solutions $x_i^{(1)}$ and $y_i^{(1)}$ where the amplitudes of these solutions depend on the output of the second level ($w_i^{(2)}$). Note that the amplitude of a sine wave, i.e. its contribution to the final output, is effectively zero while the corresponding RA ensemble in the second level is inactive (i.e., $v_i^{(2)} \approx 0$). In this way, if an RA neuron is active at the second level, the corresponding sine function contributes to the final output.

Sensitivity Analysis

Here, we investigate the effects of choosing different connectivity matrices at the third and second levels of the generative and recognition models. In the "Ideal Communication" simulation, we have used:

$$\rho_{ij} = \begin{cases} 0 & j = i, \\ 1.5 & j = i+1, \\ 0.5 & j = i-1, \\ 1 & otherwise \end{cases}$$

(Here $i+1=1$ when $i=N$ and $i-1=N$ when $i=1$) to create the HVC dynamics and used the same connectivity matrix for the recognition as well. Similar dynamics can also be obtained if the entries 1.5 and 0.5 in the above connectivity matrix are changed. In the simulation shown in Figure S4, we used 1.8 and 0.2, respectively, for these entries in both generation and recognition. Similarly, the dynamic behavior of the model is also robust to the changes at the second level. The connectivity matrix W at this level which satisfies the condition in Theorem 1 can be chosen arbitrarily as the following matrix:

$$W_1 = \begin{pmatrix} -0.7634 & -0.6390 & -0.2995 & 0.9744 & 0.5732 \\ 0.5654 & -0.0437 & 0.4338 & -0.8218 & -0.5850 \\ 0.5896 & -0.0325 & -0.6591 & 0.7162 & 0.9779 \\ -0.8933 & 0.5817 & -0.6501 & -0.2264 & -0.1209 \\ -0.9154 & -0.2407 & -0.4713 & -0.4636 & -0.5809 \end{pmatrix}$$

W_1 is used during all the simulations in the manuscript (for both generation and recognition) except the ‘Differently Wired Brains” simulation where a different matrix is used for the recognition. To show that the obtained dynamics do not change, we also changed this connectivity matrix with another arbitrary matrix that satisfies the condition of Theorem 1:

$$W_2 = \begin{pmatrix} -0.9325 & 0.8345 & -0.8415 & -0.5558 & 0.2149 \\ 0.8703 & -0.8299 & -0.8011 & 0.7111 & 0.3601 \\ 0.8152 & 0.6341 & -0.8081 & 0.9685 & 0.7585 \\ -0.2917 & -0.1723 & -0.2294 & -0.1480 & -0.7595 \\ -0.4141 & -0.5107 & -0.9282 & 0.9574 & -0.5053 \end{pmatrix}$$

As can be seen from Figure S4, the same dynamics can still be obtained with this matrix and both the generation and recognition models are robust to changes in connectivity matrices.

References

1. Matsuoka, K., *Stability Conditions for Nonlinear Continuous Neural Networks with Asymmetric Connection Weights.* Neural Networks, 1992. **5**(3): p. 495-500.
2. Zheng, P.S., W.S. Tang, and J.X. Zhang, *Efficient Continuous-Time Asymmetric Hopfield Networks for Memory Retrieval.* Neural Computation, 2010. **22**(6): p. 1597-1614.
3. Friston, K.J., N. Trujillo-Barreto, and J. Daunizeau, *DEM: A variational treatment of dynamic systems.* Neuroimage, 2008. **41**(3): p. 849-885.
